# Sustainable Ir-Photoredox Catalysis by Means of Heterogenization

**DOI:** 10.1021/acsorginorgau.2c00024

**Published:** 2022-08-02

**Authors:** Rickard Lindroth, Kelly L. Materna, Leif Hammarström, Carl-Johan Wallentin

**Affiliations:** † Department of Chemistry and Molecular Biology, University of Gothenburg, Gothenburg SE41296, Sweden; ‡ Department of Chemistry-Ångström Laboratories, Uppsala University, Box 523, Uppsala SE75120, Sweden

**Keywords:** heterogenized photocatalyst, sustainable photocatalysis, reusable, iridium, photoredox catalysis, sustainable photoredox

## Abstract

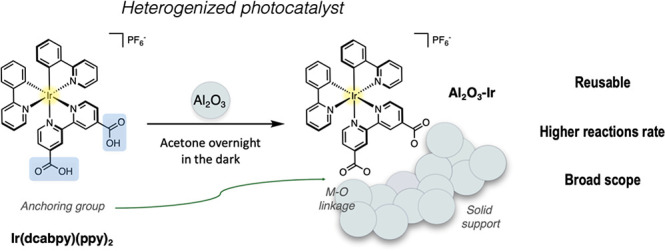

A heterogenized iridium catalyst was employed to perform photoredox catalysis for a collection of mechanistically orthogonal reactions using very low quantities of iridium (0.01–0.1 mol %). The heterogenized construct consists of an organometallic iridium coordination complex bonded to an aluminum metal oxide solid-state support via an anchoring group. The solid-state support allows for easy recovery and reusability of the catalyst. Evaluation of the catalytic activity was performed with five different reactions, showing broad applicability and demonstrating the general potential for a heterogenized strategy. Moreover, the heterogenized catalyst was shown to be reusable up to five times and also mediated the reactions with much higher efficiency than the original processes by employing the corresponding homogeneous catalyst. As a result of the low catalyst loadings employed, the feasibility of reusage, and faster reaction times, this catalyst offers a more sustainable option when precious metal catalysts are used in organic synthesis. Finally, the catalyst was successfully applied to a gram-scale reaction, showing it is susceptible to scalability.

The field of photoredox catalysis has emerged as a prominent contributor to a greener production of chemicals by harvesting visible light as an energy source.^1−6^ Photoredox catalysts operate by enabling reactions via energy transfer or the generation of radical intermediates under mild conditions without the use of harsh reagents or high temperatures traditionally associated with radical generation. Typically, homogeneous solution-phase catalysts based on organometallic coordination complexes are employed, where derivatives of tris(2,2′-bipyridine)ruthenium or tris(2-phenylpyridine) iridium represent some prevalent catalyst structures. Upon irradiation, these catalysts undergo a metal-to-ligand charge-transfer transition followed by intersystem crossing, generating long-lived excited states (ns−μs) that are concurrently strongly oxidizing and reducing, compared to their ground-state redox potentials. Such constitutional properties allow them to perform a plethora of organic transformations. Nonetheless, a prospective large-scale employment of precious metals is not a sustainable conception.

Efforts to expand the catalyst repertoire beyond precious metals has the focus on the development of organic and earth-abundant metal photocatalysts. These strategies, however, are associated with caveats in terms of shorter excited state lifetimes and catalyst stability, limiting a broad applicability and their use in sustainable upscale contexts. Recently, we have begun exploring *heterogenized* photoredox catalysts, which maintains the advantageous properties of precious metal catalysts, and developed a catalytic material that consists of photocatalysts immobilized on metal oxide solid-state supports.^7^ The catalysts are attached via a surface-anchoring group (e.g., carboxylic acids, phosphonic acids, hydroxamic acids, or silatranes), a strategy commonly employed in the solar fuel field for water oxidation, hydrogen production, and carbon dioxide reduction (Figure 1).^8−15^ This heterogenization of photocatalysts allows for recovery and reusage of the catalysts by simple filtration or centrifugation: a feasible separation technique compatible with scale-up. In spite of this strategy being extensively utilized in the solar field, limited work has been done in the field of photoredox catalysis with only a few examples reported using a heterogenized strategy.^7,16−24^

**Figure 1:**
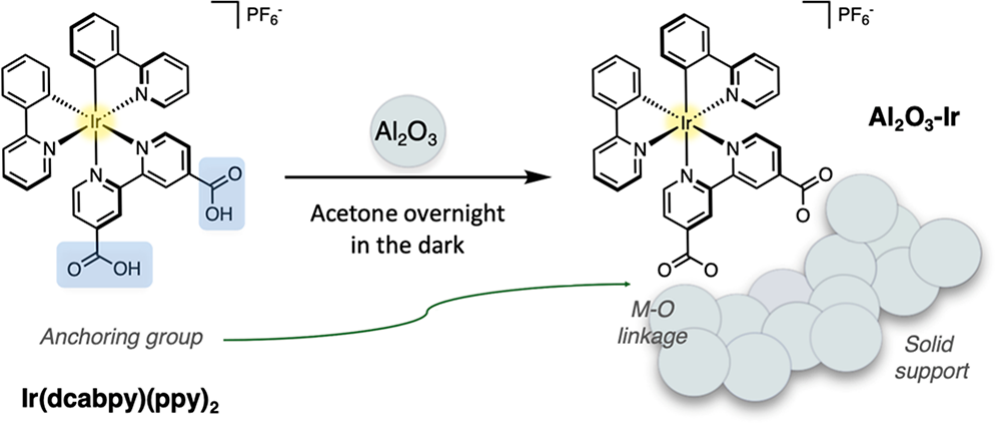
Heterogenization of Ir(dcabpy)(ppy)_2_ (left) by anchoring onto aluminum oxide. Al_2_O_3_–Ir (right) is formed when carboxylic acid surface anchors are stirred in a suspension of Al_2_O_3_ nanopowders in acetone overnight.

Previously, a systematic study from our group has generated a design strategy of several heterogenized catalyst architectures to determine which construct was optimal for photoredox catalysis.^7^ The catalyst Ir(dcabpy)(ppy)_2_, a bis(2-phenylpyridine)(2,2′-bipyridine)iridium coordination complex, which employed two carboxylic acids as surface anchors on the bipyridine ligand, was synthesized (Figure 1). The effect of the metal oxide solid-state support was explored in terms of composition [Al_2_O_3_, ZrO_2_, and indium tin oxide (ITO)] and architecture (nanopowders or thin films). The insulating support materials (i.e., Al_2_O_3_ and ZrO_2_) are redox-inactive during catalysis, causing no annihilative electron transfers, whereas the conductive material ITO is more suitable in future photoelectrochemical (PEC) setups, removing any need for sacrificial reagents. Nanopowder architectures were deemed better suited for performing synthetic reactions as they were conveniently utilized in agitated batch reactors, and film architecture was deemed more suitable for applications in PEC setups. Promising results were observed for the reductive dehalogenation reaction of 2-bromoacetophenone to acetophenone.^25^ The best performing and most robust catalyst for this model reaction was the nanopowder based on Al_2_O_3_ (>1000 TON), completing the reaction in 15 min.

In light of these observations, we wanted to explore the reaction scope and applicability of the catalyst of our Al_2_O_3_-based nanopowder catalyst Al_2_O_3_–Ir (Figure 1). Herein, we report on a study of five different reaction classes (Figure 2), targeting catalyst engagement in both oxidative and reductive quenching pathways as well as mechanistically distinct energy transfer. The Al_2_O_3_–Ir catalyst operates efficiently under many conditions and at very low catalyst loadings (down to 0.01 mol %). Furthermore, we show that the heterogenized catalyst is more efficient compared to the homogeneous congener and can be employed for gram-scale synthesis. Finally, we emphasize that the reusability, low catalysts loadings, and scalability render the heterogenized constructs qualified as a more economical competitive alternative to both organic and earth-abundant catalysts.

**Figure 2:**
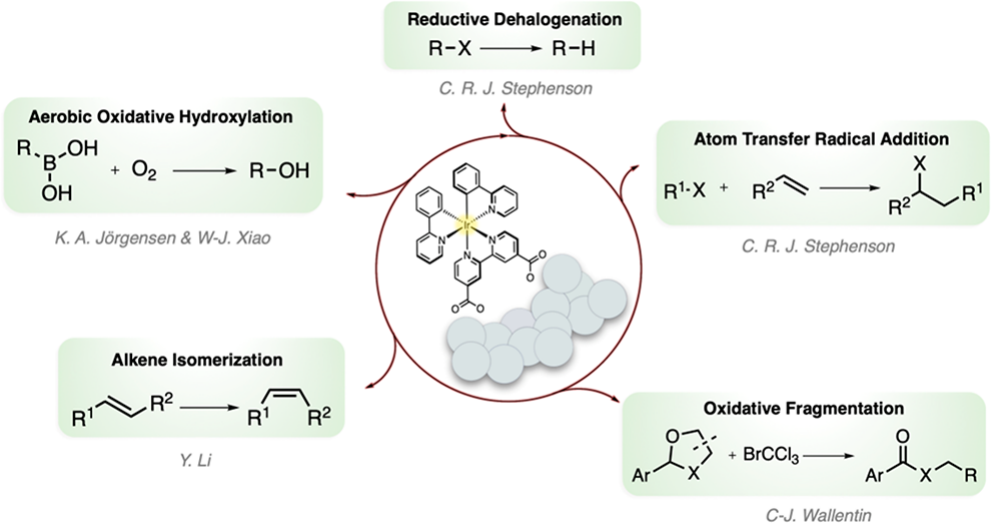
Five reaction classes examined using the heterogenized catalyst Al_2_O_3_–Ir.

## Results and Discussion

For full exploration of the utility of the heterogenized catalyst, four reactions that constitute examples of both oxidative and reductive quenching mechanisms were selected. These include reductive dehalogenation,^25^ atom-transfer radical addition (ATRA),^26^ oxidative fragmentation of ethers and acetals,^27^ and aerobic oxidative hydroxylation of boronic acids^28^ (Figure 2). Additionally, we also explored the olefin *E*-to-*Z* isomerization of cinnamates to evaluate the performance of an energy-transfer pathway from the excited triplet state of the catalyst, *PC(T_1_), to generate the excited triplet state of the alkene.^29^

### Catalyst Stability in Various Solvents

As catalyst instability can be a major issue in heterogenized systems, a solvent stability study was initially performed to determine in what solvent systems the catalyst remained intact. Catalyst desorption from metal oxide surfaces is a frequent issue for heterogenized catalysts and constitute a mode of catalyst instability.^30−32^ If catalyst desorption occurs, then not only does a homogeneous catalyst possibly contribute to the acceleration of the reaction but also a less potent heterogeneous catalyst will be recovered, ultimately wasting precious iridium. We wanted to ensure that the stability of Al_2_O_3_–Ir in our solvent systems remains heterogenized during catalysis by analyzing the desorption kinetics with UV–vis spectroscopy.

The following common solvents were screened: dichloromethane, methanol, dimethylformamide, dimethyl sulfoxide, acetonitrile, ethyl acetate, chloroform, tetrahydrofuran, pentane, toluene, 4:3 methanol/acetonitrile, 1,2-dichloroethane (DCE), and water. Al_2_O_3_–Ir was soaked in deoxygenated solvent under irradiation for 2 h. Thereafter, the catalyst was separated by centrifugation, the supernatant was filtered through a fine frit filter, and UV–vis spectra of the solutions were recorded. Ir(dcabpy)(ppy)_2_ has a signature UV–vis absorption band at 370 nm, which would appear in the spectrum if Ir(dcabpy)(ppy)_2_ had desorbed from the Al_2_O_3_ surface.^7^ Acetonitrile, methanol, water, chloroform, and dimethyl sulfoxide caused a significant desorption of Ir(dcabpy)(ppy)_2_. The solvents causing no or little desorption were dichloromethane, dimethylformamide, ethyl acetate, tetrahydrofuran, pentane, toluene, DCE, and unexpectedly 4:3 methanol/acetonitrile (see the Supporting Information). Having a good grasp of compatible solvent systems, we next proceeded to explore the five reactions.

### Reductive Dehalogenation

**Scheme 1:**
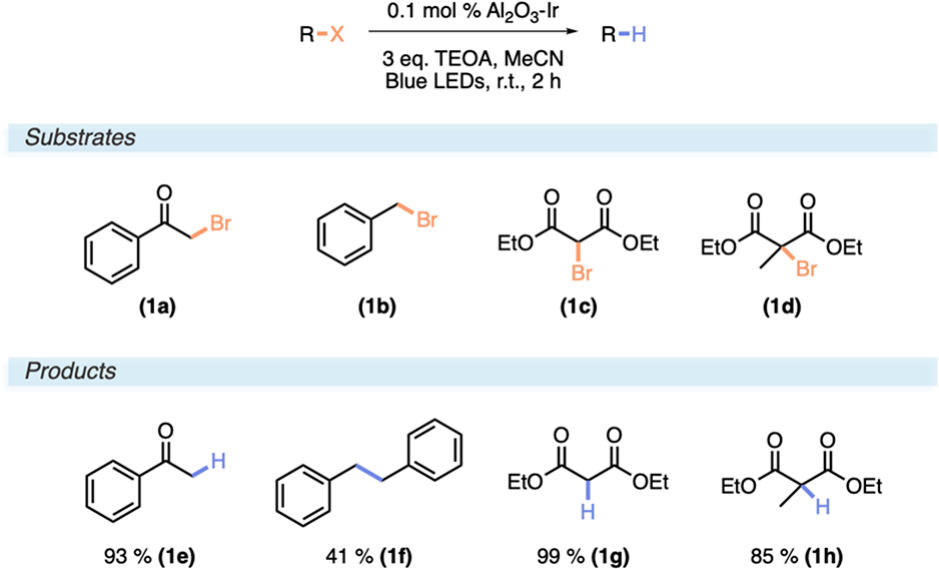
Reaction Conditions and Substrate Scope for the Reductive Dehalogenation

This reaction has previously been explored by the catalytic construct and been shown to rely on triethanolamine (TEOA) as a stoichiometric reductant (*vide supra*). Further optimization of the reductive dehalogenation of 2-bromoacetophenone (**1a**) to acetophenone (**1e**) showed that as low as 0.1 mol % Al_2_O_3_–Ir efficiently catalyzed the reaction to 93% yield with only 2 h of irradiation (Scheme 1). Expansion of the substrate scope beyond 2-bromoacetophenone to benzyl bromide (**1b**) proved successful in forming a dibenzyl dimer (**1f**) in 41% yield rather than toluene. Moreover, both diethyl-2-bromomalonate (**1c**) and diethyl-2-bromo-2-methylmalonate (**1d**) were found to convert to completion in 2 h, forming diethyl malonate (**1g**) and diethyl-2-methylmalonate (**1h**), respectively.

### Atom-Transfer Radical Addition (ATRA)

**Scheme 2:**
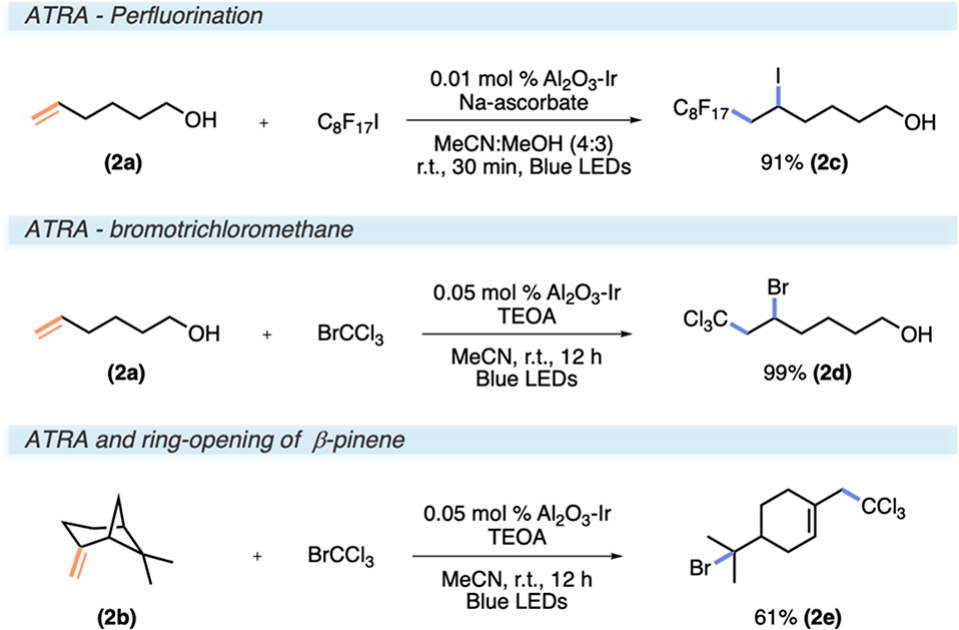
Reaction Conditions Substrate Scope for ATRA Reactions

Next, we proceeded with a set of three ATRA reactions that were developed for both oxidative and reductive quenching mechanisms, as reported by Stephenson.^26^ In some cases, an oxidative quenching of either Ru(bpy)_3_ or [Ir{dF(CF_3_)ppy)_2_}(dtbbpy)]^+^ sufficed to promote a reaction; in other cases, a sacrificial electron donor, either stoichiometrically or substoichometrically, was needed. Mechanistically, the reaction is initiated via a SET to a halide substrate, which promotes a mesolytic cleavage, giving a carbon-centered radical and a halide anion. The radical typically adds to an alkene, and the halide anion captures the carbocationic intermediate formed upon the photocatalyst oxidizing the radical intermediate. The reaction is postulated to also include propagating features. The first reaction explored constituted a perfluorination of 5-hexene-1-ol that was successfully reproduced with the same conditions, yielding 91% (**2c**), however with a catalyst loading of only 0.01 mol % Al_2_O_3_–Ir (Scheme 2). The other two focusing on the ATRA of BrCCl_3_ to 5-hexene-1-ol and β-pinene could not be reproduced directly by substitution of the catalyst. With the addition of TEOA as a reductive quencher, the other two ATRA reactions proceeded smoothly to give ATRA product **2d** in 99% yield and the β-pinene derivative **2e** in 61% yield. The perfluorination was also conducted at a gram scale, providing a 99% isolated yield.

### Aerobic Oxidative Hydroxylation of Boronic Acids

**Scheme 3:**
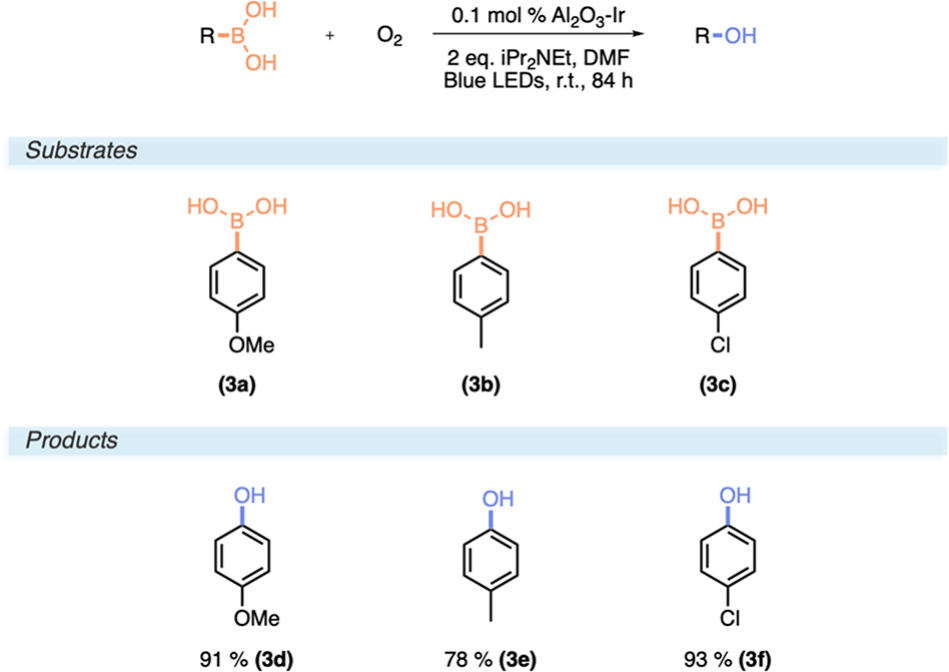
Reaction Conditions and Substrate Scope for the Aerobic Oxidative Hydroxylation of Boronic Acids

Next, we applied the catalyst to the oxidative hydroxylations of aryl boronic acids giving phenols. These reactions use *N*,*N*-diisopropylethylamine (Hünig’s base) as a sacrificial reductive quencher of *Ru(bpy)_3_^2+^ that proceeds to reduce molecular oxygen from the surrounding air to give the superoxide radical anion O_2_^–^, which ultimately functions as the oxidant.^28^ The O_2_^–^ reacts with the boron atom, and after a hydrogen atom transfer (HAT) involving the radical cation of Hünig’s base, a rearrangement and, after hydrolysis, a phenol are generated. We screened three para-substituted derivatives of phenylboronic acid (methoxy, methyl, and chloro) using 0.1 mol % Al_2_O_3_–Ir and 84 h of irradiation in dimethylformamide (Scheme 3). All substrates produced the phenol products in high yields ranging from 78% for **3e** to 93% for **3f**. Although we increased the reaction times three- to fourfold, depending on the substrate, we were able to go down to 5% of the reported catalyst loading (0.1 mol % Al_2_O_3_–Ir vs 2% Ru(bpy)_3_).^28^

### Oxidative Fragmentation of Ethers and Acetals

**Scheme 4:**
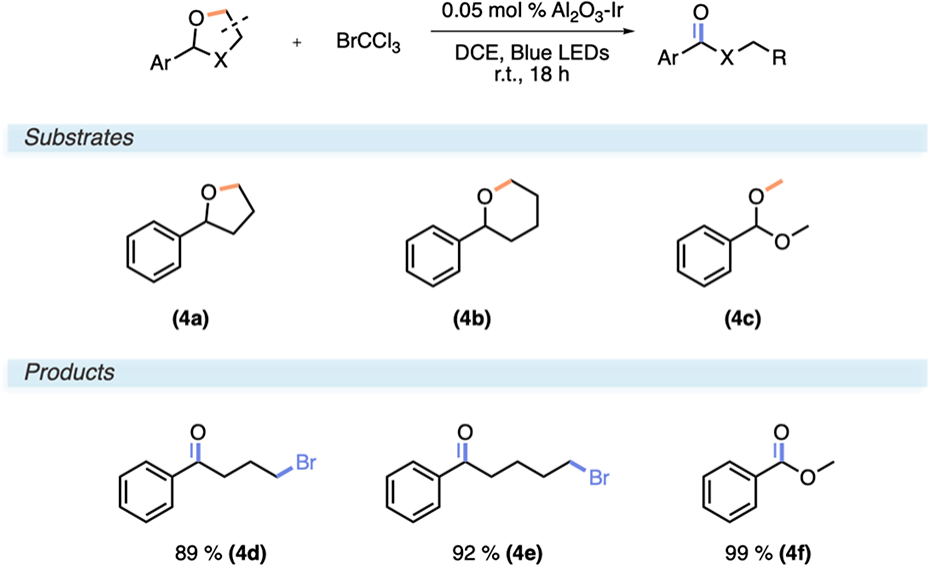
Reaction Conditions and Substrate Scope for Oxidative Fragmentation of Ethers and Acetals

We have recently in our laboratory disclosed an Fe-catalyzed oxidative fragmentation of ethers and acetals giving distally brominated ketones or esters, respectively (Scheme 4).^27^ Similar to that in the ATRA reactions, the catalyst engages BrCCl_3_ reductively, giving a trichloromethyl radical and a bromide ion after mesolytic cleavage. But contrary to an addition, the trichloromethyl radical abstracts a hydrogen from the substrate to generate an α-oxyradical. After a bromine abstraction from BrCCl_3_, an intramolecular expulsion of Br^–^ provides the products after a nucleophilic attack of Br^–^. Somewhat surprisingly, BrCCl_3_ could directly be reduced without the addition of a sacrificial electron donor, contrary to some ATRA examples. 2-Phenyltetrahydrofuran (**4a**) and 2-tetrahydro-2*H*-pyran (**4b**) both underwent this oxidative fragmentation in high yields, 89% (**4d**) and 92% (**4e**), respectively, without any further modification of reaction conditions other than employment of 0.05 mol % Al_2_O_3_–Ir. Also, the dimethyl acetal of benzaldehyde could be converted to the corresponding methyl benzoate in excellent 99% yield (**4f**) along with the coformation of MeBr.

### *E*–*Z* Isomerization of Cinnamates

Because both oxidative and reductive quenching pathways were mediated with very high efficiency in the investigated reaction classes above, we wanted to further explore the performance of the catalyst while operating via an energy transfer. An *E*–*Z* alkene isomerization of cinnamates has been postulated to occur via a triplet energy transfer by Ir(ppy)_3_.^29^ An efficient quenching of *[Ir(ppy)_3_] by *E*-cinnamates in a singlet ground state generates an excited triplet state that either relaxes back the ground state or undergoes a rotation about the σ bond preceding relaxation. The latter constitutes a synthetically productive pathway, eventually causing an accumulation of the *Z*-isomer as it quenches the catalyst at a much lower rate than does the *E*-isomer.

**Scheme 5:**
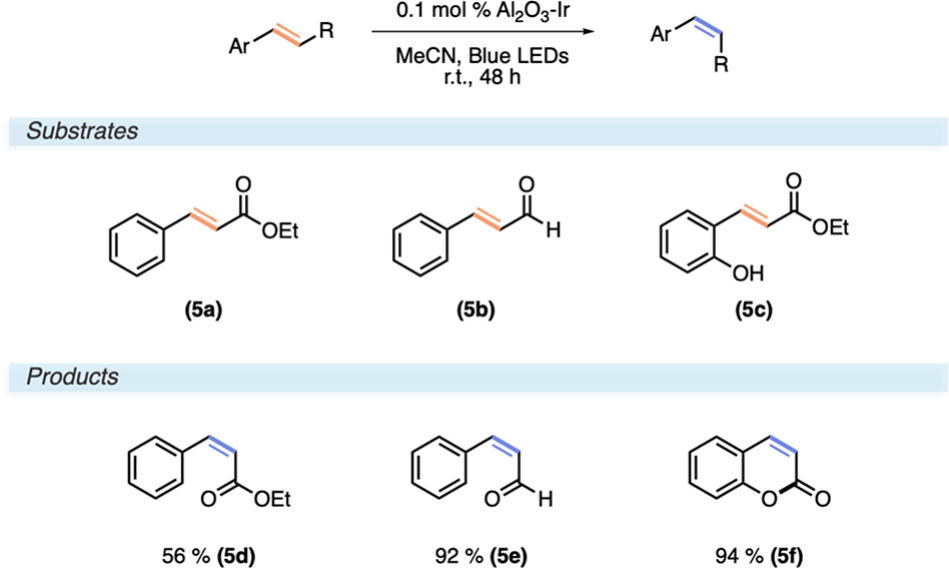
Reaction Conditions and Substrate Scope for the Isomerization of Cinnamates and Coumarin Synthesis

The two alkenes (*E*)-ethyl cinnamate (**5a**) and (*E*)-cinnamaldehyde (**5b**) underwent isomerization to the corresponding (*Z*) isomers after 48 h of irradiation in 56% and 92% yield, respectively (Scheme 5). Note, Al_2_O_3_–Ir performed the reaction with one-tenth of the catalyst loading to the homogeneous Ir(ppy)_3_, as reported by Li and Zhan.^29^ Although we doubled the reaction time, the yield is nearly twice as high for **6b** compared to the previously reported yield of 54% for this compound.

This chemistry was further applied to the synthesis of coumarins via lactonization with the *o*-OH substituent of 2-hydroxycinnamates after the isomerization. Coumarins are important dyes and find applications in dye-sensitized solar cells (DSSCs) and water-splitting DSSCs to produced renewable electricity and fuels, respectively.^33,34^ Indeed, ethyl-2-hydroxicinnamate (**6c**) could be converted to the coumarin 2*H*-chromen-2-one (**6f**) with a similar excellent yield of 94%.

### Comparing the Rate of Reaction for Heterogenized and Homogeneous Catalysts

**Figure 3:**
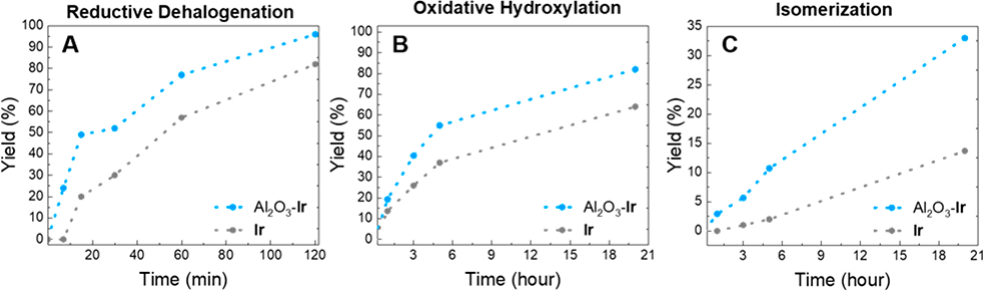
Reaction yields against time for the heterogenized Al_2_O_3_–Ir catalyst (blue) and the homogeneous Ir(dcabpy)(ppy)_2_ (gray). Dehalogenation of 2-bromoacetophenone (A), oxidative hydroxylation of 4-chlorophenylboronic acid (B), and isomerization of (*E*)-ethyl cinnamate (C). 0.1 mol % was used for all reactions, where were conducted in deuterated solvent. Yields were monitored by ^1^H NMR with an internal standard. **Ir** in the graphs refers to Ir(dcabpy)(ppy)_2_.

As we noted in our previous work,^7^ Al_2_O_3_–Ir catalyzed the dehalogenation of 2-bromoacetophenone faster than the homogeneous Ir(dcabpy)(ppy)_2_. Further examination of this intrinsic property of the heterogenized catalyst was carried out by monitoring the reactions over time. We chose to study the rate of three reactions: the reductive dehalogenation of 2-bromoacetophenone (**1a**), the oxidative hydroxylation of 4-chlorophenylboronic acid (**3c**), and the alkene isomerization of (*E*)-ethyl cinnamate (**5a**). In all examples, the reaction rate of the heterogenized catalyst was faster than that of the homogeneous version (Figure 3). From the figure it is clear that Al_2_O_3_–Ir gives a higher yield at each point in time. For the dehalogenation a much higher initial rate is observed that, however, slows down and reaches a similar rate of reaction after 30 min. The same trend is observed for the oxidative hydroxylation with a significantly higher initial rate. For the isomerization, however, the higher reaction rate was observed throughout the entire reaction time with a relatively steady rate, yielding a greater difference in yield of product after 20 h compared to those seen in the former examples.

To account for these observations, we propose some possible factors that are likely to play a significant role in allowing the heterogenized catalyst to perform the reactions more quickly. (1) Al_2_O_3_ is often used as a support material in heterogeneous catalysis because of its ability to adsorb reactants efficiently. If the adsorption of reactants is favorable, it gives rise to a higher local concentration of reactants that effectively increase the reaction rate. (2) Al_2_O_3_ is also a Lewis acid that could act in conjunction to activate substrates by lowering the barrier for quenching of the photocatalyst. (3) Because of the nature of heterogenized constructs, any reactions or interactions between photocatalysts are suppressed. Note that for homogeneous photocatalysts there is always the possibility for self-quenching, which may effectively be eliminated when the catalysts are immobilized onto an Al_2_O_3_ support, provided the loading density is not too high. Thus, the lower possibility for self-quenching in heterogenized supports could help increase the reaction yields. (4) It is possible that the heterogenized catalyst changes the mechanism of the reaction, as observed previously for water oxidation with an iridium catalyst attached to an ITO surface.^9^ In this example the heterogenized catalyst operated via a monomeric mechanism, whereas in the homogeneous case a dimeric iridium complex that changed the mechanism was formed. It has also previously been reported that a heterogenized construct could prevent catalyst degradation pathways such as ligand dissociation/degradation.^35−37^ Thus, altogether, these points may explain why the heterogenized catalysts give a higher yield over time compared to the homogeneous ones.

### Reusability Tests

Because precious metal catalysts are both expensive and rare, we wanted to explore to what extent our heterogenized catalyst can be reused. Accordingly, we investigated the reusability of a 0.1 mol % loading of the Al_2_O_3_–Ir construct by recycling the catalyst five times sequentially for the reductive dehalogenation reaction (Figure S2). Indeed, we found that the catalyst was recyclable, with yields staying at nearly 80% for up to four cycles, and dropping somewhat to 62% after the fifth cycle. Next, the oxidative hydroxylation of 4-chlorophenylboronic acid (**3c**) and isomerization of (*E*)-ethyl cinnamate (**5a**) were evaluated. Both reaction classes were found to generate product over three cycles. However, the oxidative hydroxylation reactions dropped from 93% to 50% yield and the alkene isomerization from 56% to 25% yield after the last cycle.

Some differences in the ability to reuse the catalysts were observed as catalyst deterioration is affected by parameters such as solvent, irradiation time, and the chemical environment around the catalyst. The oxidative hydroxylation and alkene isomerization were conducted for much longer periods [5 and 20 h, respectively (Figure 3)] compared to the reductive dehalogenation (30 min). A protective layer of alumina using atomic layer deposition could potentially be used in future efforts to provide more stable catalysts; this has been shown to be an effective strategy in the solar fuels field.^38,39^ Regardless, the catalyst remained catalytically active for recycling up to five times for the reactions tested, which demonstrate the potential for heterogenization strategies.

In conclusion, we have demonstrated a broad applicability of the heterogenized iridium photoredox catalyst, Al_2_O_3_–Ir. Four reactions operating via both oxidative and reductive quenching pathways and one energy-transfer-mediated reaction were evaluated and demonstrated to progress efficiently. The catalysts can be used with very low catalysts loadings (0.01 mol %) compared to many reported homogeneous catalysts. The catalysts were reusable up to five times, creating less waste of iridium, emphasizing their potential to provide a more economical option for precious metal catalysis. Additionally, taken together with the demonstration of a successful gram-scale reaction, we want to highlight the legitimacy of a heterogenized strategy in potential future industrial applications. Furthermore, we emphasize that these reactions can be performed with minimal quantities of iridium to obtain similar yields to the homogeneous catalysts commonly employed in photoredox catalysis. As a considerate consumption of precious metals is a sustainable conception, we hope to spur further research in this area. To conclude, we believe heterogenized iridium constructs makes for a good step toward sustainable photoredox catalysis involving precious metals.

## Supporting Information Available

Experimental procedures and characterization (PDF)
