# Supplementary material for: Sustainable Ir-Photoredox Catalysis by Means of Heterogenization
Source: ACS Org Inorg Au. 2022 Aug 2;2(5):427–32. doi: 10.1021/acsorginorgau.2c00024 (PMC9955341; doi:10.1021/acsorginorgau.2c00024)

Supporting Information

Sustainable Ir-Photoredox Catalysis by Means of Heterogenization

Rickard Lindroth[a], Kelly L. Materna*[b], Leif Hammarström[b], Carl-Johan Wallentin[a]*

[a] Department of Chemistry and Molecular Biology, University of Gothenburg, SE-412 96, Gothenburg, Sweden

[b]Department of Chemistry-Ångström Laboratories, Uppsala University, Box 523, SE-751 20, Uppsala, Sweden.

***Corresponding authors:** kelly.l.materna@gmail.com, carl.wallentin@chem.gu.se

General information

All reagents and solvents were purchased from Merck, Sigma-Aldrich or VWR. All chemicals were used as received. All reactions were performed under an atmosphere of argon. TLC plates (Silica gel 60 F_{254} coated on aluminum plates purchased from Sigma-Aldrich and VWR) were visualized by either UV light (254 nm) or $KMnO_4$ solution. Flash column chromatography was performed both manually (using Silica gel 40-63 µm from VWR) and with Büchi Pure C-810 Flash (using Büchi FlashPure EcoFlex cartridges 50 µm).

GC-MS(EI) was performed on Agilent 19091S-433 gas chromatograph with an Agilent 5977E MSD detector or an Agilent 8890 GC / 5977B GC/MSD.

A Varian Cary-50 Bio spectrophotometer was used to perform UV-Vis spectroscopic measurements.

^{1}H-NMR and ^{13}C-NMR spectra were recorded on a Varian NMR 400 or A JEOL (400YH magnet) Resonance 400 MHz spectrometer. Chemical shifts (δ) are reported in ppm relative to the residual solvent peak. Splitting patterns are indicated as (s) singlet, (d) doublet, (t) triplet, (q) quartet. Coupling constants (*J*) are reported in Hertz (Hz).

Preparation of Al_2O_3-Ir catalyst

The Al_2O_3-Ir catalyst was prepared by first synthesizing Ir(dcabpy)$(ppy)_2$. 2.5 mL of a 0.25 mM solution of Ir(dcabpy)$(ppy)_2$ in acetone was stirred in the presence of 25 mg of Al_2O_3 (aluminum oxide nanopowder (<50 nm)) in the dark overnight. The catalyst was centrifuged and rinsed with acetone three times followed by drying under vacuum overnight. The amount of Ir(dcabpy)$(ppy)_2$ linked to the metal oxide surface was determined with UV-vis spectroscopy by quantifying the concentration of Ir(dcabpy)$(ppy)_2$ in the solution before and after reaction with Al_2O_3 using the signature peak at 370 nm, extinction coefficient of 8737 M^{-1} cm^{-1},[1] of Ir(dcabpy)$(ppy)_2$. Ir(dcabpy)$(ppy)_2$ was found to bind a loading of 16 ± 0.6 nmol/mg on average. Full characterization of the catalyst can be found in the prior study *Chem. Eur. J.* **2021**, 27, 16966-16977.

General Procedures

All Photoreactions were conducted in Biotage® Microwave Reaction Vials (10 ml) placed in EvoluChem™ PhotoRedOx Box By HepatoChem equipped with a Kessil lamp (A160WE) as irradiation source. Reactions were cooled with a fan in the photoreactor (27 ^{o}C). Needle holes after degassing were covered with puddy (Play-Doh) or parafilm.

1. Reductive dehalogenation

Bromo compound (0.1 mmol), Al_2O_3-Ir (100 nmol, 0.1 mol%), and triethanolamine (0.3 mmol (3 eq.) were mixed in anhydrous MeCN (1 ml). The reactions were sparged with Ar for 10 min and irradiated for 2h. Reaction mixture was adsorbed onto Celite prior to purification on silica.

2. Atom transfer radical addition (ATRA)

For ATRA with $BrCCl_3$: Alkene (0.1 mmol), $BrCCl_3$ (0.2 mmol, 2 eq.), TEAO (0.3 mmol, 3 eq.) and Al_2O_3-Ir (50 nmol, 0.05 mol% Ir) and anhydrous MeCN (1 ml) were mixed and sparged

with Ar 10 min. Reactions were irradiated with for 12 h. Reaction mixture was adsorbed onto Celite prior to purification on silica.

For perfluorination: 5-hexene-1-ol (0.1 mmol), $C_8F_{17}I$ (0.13 mmol, 1.3 eq.), Na-ascorbate (0.035 mmol, 0.35 eq.), Al_2O_3-Ir (10 nmol, 0.01 mol%), MeCN (0.8 ml) and MeOH (0.6 ml) were mixed and sparged with Ar 10 min. Reactions were irradiated with for 30 min. Reaction mixture was adsorbed onto Celite prior to purification on silica.

3. Aerobic oxidative hydroxylation of boronic acids

Boronic acid (0.1 mmol), Al_2O_3-Ir (100 nmol, 0.1 mol%), N,N-diisopropylethylamine (0.2 mmol, 2 eq.) and anhydrous DMF (1 ml) and stirred open to air under irradiation for 84 hours. Crude was gently concentrated under reduced pressure and then adsorbed onto Celite, removing the residual DMF during purification on silica.

4. Oxidative fragmentation of ethers and acetals

Cyclic ether or acetal (0.1 mmol), $BrCCl_3$ (0. 3 mmol, 3 eq.), Al_2O_3-Ir (50 nmol, 0.05 mol%) and DCE (1ml) were mixed and sparged with Ar 10 min. Reactions were irradiated for 18 h.

For benzaldehyde dimethyl acetal: Reaction was filtered over a short silica plug and eluted with DCM, concentrated under reduced pressure to afford pure methyl benzoate.

For cyclic ethers: Reaction mixture was adsorbed onto Celite prior purification on silica.

5. Alkene isomerization

(E)-alkene (0.1 mmol), Al_2O_3-Ir (100 nmol, 0.1 mol%) and MeCN (1 ml) were mixed and sparged with Ar 10 min and irradiated for 48 hours. Reaction mixture was adsorbed onto Celite and purified on silica (20 μm) to separate the isomers.

Product Characterizations

I. Reductive dehalogenation

Acetophenone (1e). Performed according to general procedure 1. Colorless liquid (11 mg, 93 %). Purified on silica with 10 % Et_2O in pentane (R_f =0.48). **^{1}H NMR** (400 MHz, $CDCl_3$) δ 8.00 – 7.92 (m, 2H), 7.61 – 7.52 (m, 1H), 7.51 – 7.41 (m, 2H), 2.60 (s, 3H). **^{13}C{^{1}H} NMR** (101 MHz, $CDCl_3$) δ 198.2, 137.1, 133.1, 128.6, 128.3, 26.6. **MS(EI)**: Calculated for $C_8H_8O^+$ 120.0570; found 120.1.

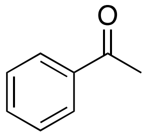

Bibenzyl (1f). Performed according to general procedure 1. Colorless solid (4 mg, 41%). Purified on silica with 2 % EtOAc in hexane (R_f = 0.4). **^{1}H NMR** (400 MHz, $CDCl_3$) δ 7.37 – 7.26 (m, 4H), 7.25 – 7.16 (m, 6H), 2.94 (s, 4H). **^{13}C{^{1}H} NMR** (101 MHz, $CDCl_3$) δ 141.8, 128.5, 128.3, 125.9, 38.0. **MS(EI)**: Calculated for $C_{14}H_{14}^+$ 182.1096; found 182.1.

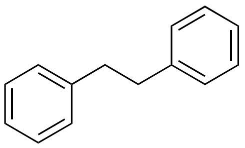

Diethyl malonate (1g). Performed according to general procedure 1. Colorless oil (16 mg, 99 %). Filtered through a short silica plug, eluted with Et_2O. **^{1}H NMR** (400 MHz, $CDCl_3$) δ 4.21 (q, *J* = 7.1 Hz, 4H), 3.36 (s, 2H), 1.28 (t, *J* = 7.1 Hz, 6H). **^{13}C{^{1}H} NMR** (101 MHz, $CDCl_3$) δ 166.6, 61.5, 41.7, 14.0. **MS(EI)**: Calculated for $C_7H_{12}O_4^+$ 160.0730; found 160.1.

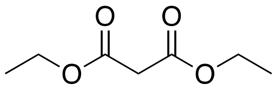

Diethyl methylmalonate (1h). Performed according to general procedure 1. Colorless oil (15 mg, 85 %). Filtered through a short silica plug, eluted with Et_2O (care should be taken as **1h** one turned out to be very volatile). **^{1}H NMR** (400 MHz, $CDCl_3$) δ 4.19 (qd, *J* = 7.1, 2.2 Hz, 4H), 3.41 (q, *J* = 7.3 Hz, 1H), 1.41 (d, *J* = 7.2 Hz, 3H), 1.26 (t, *J* = 7.1 Hz, 6H). **^{13}C{^{1}H} NMR** (101 MHz, $CDCl_3$) δ 170.2, 61.3, 46.2, 14.0, 13.5. **MS(EI)**: Calculated for $C_8H_{14}O_4^+$ 174.0887; found 174.1.

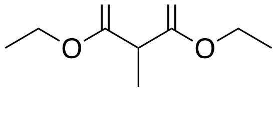

II. Atom transfer radical addition (ATRA)

7,7,8,8,9,9,10,10,11,11,12,12,13,13,14,14,14-heptadecafluoro-5-iodotetradecan-1-ol (2c). Performed according to general procedure 2. Colorless oil (59 mg, 91 %). Purified on silica with 100 % DCM as eluent (R_f = 0.17). **^{1}H NMR** (400 MHz, $CDCl_3$) δ 4.34 (tdd, *J* = 8.4, 5.4, 4.3 Hz, 1H), 3.69 (t, *J* = 6.0 Hz, 2H), 3.03 – 2.69 (m, 2H), 1.90 – 1.74 (m, 2H), 1.70 – 1.45 (m, 4H). **^{13}C{^{1}H} NMR** (101 MHz, $CDCl_3$) δ 122.0 – 104.5 (m), 62.3, 41.6 (t, *J* = 20.9 Hz), 40.0 (d, *J* = 2.2 Hz), 31.4, 26.0, 20.4. **MS(EI)**: Calculated for $C_{14}H_{12}F_{17}IO^+$ 645.9656; found 519.2 [(M-I)$^+$].

For large scale reaction **2c** was obtained as a colorless oil (1.28 g, 99%).

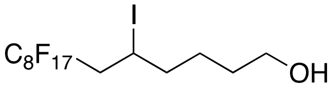

5-bromo-7,7,7-trichloroheptan-1-ol (2d). Performed according to general procedure 2. Colorless oil (30 mg, 99 %). Purified on silica with 2 % EtOAc in petroleum ether as eluent (R_f = 0.21). **^{1}H NMR** (400 MHz, $CDCl_3$) δ 4.34 (dq, *J* = 9.4, 4.9 Hz, 1H), 3.77 – 3.59 (m, 2H), 3.51 – 3.19 (m, 2H), 2.14 – 1.93 (m, 2H), 1.68 – 1.56 (m, 5H). **^{13}C{^{1}H} NMR** (101 MHz, $CDCl_3$) δ 97.1, 62.6, 62.5, 48.9, 39.2, 31.7, 23.6. **MS(EI)**: Calculated for $C_7H_{12}BrCl_3O^+$ 295.9132; did not ionize.

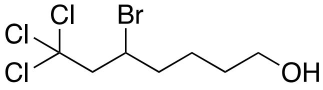

4-(2-bromopropan-2-yl)-1-(2,2,2-trichloroethyl)cyclohex-1-ene (2e). Performed according to general procedure 2. Colorless oil (20 mg, 61 %). Purified on silica with 100 % petroleum ether as eluent (R_f = 0.34). **^{1}H NMR** (400 MHz, $CDCl_3$) δ 5.80 (p, *J* = 2.4 Hz, 1H), 3.38 – 3.28 (m, 2H), 2.46 – 2.27 (m, 3H), 2.13 – 2.02 (m, 2H), 1.82 – 1.75 (m, 6H), 1.72 – 1.62 (m, 1H), 1.49 – 1.36 (m, 1H). **^{13}C{^{1}H} NMR** (101 MHz, $CDCl_3$) δ 131.2, 130.5, 99.0, 72.6, 61.9, 46.8, 32.4, 31.8, 30.6, 28.7, 26.0. **MS(EI)**: Calculated for $C_{11}H_{16}BrCl_3^+$ 331.9495; did not ionize.

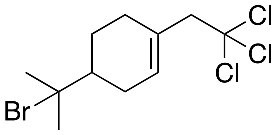

IV. Aerobic oxidative hydroxylation of boronic acids

4-Methoxyphenol (3d). Performed according to general procedure 3. White solid (11 mg, 91%). Purified on silica with 10 % Et_2O in pentane (R_f = 0.10). **^{1}H NMR** (400 MHz, $CDCl_3$) δ 6.86 – 6.70 (m, 4H), 4.78 (s, 1H), 3.77 (s, 3H). **^{13}C{^{1}H} NMR** (101 MHz, $CDCl_3$) δ 153.7, 149.4, 116.0, 114.8, 55.8. **MS(EI)**: Calculated for $C_7H_8O_2^+$ 124.0519; found 124.0.

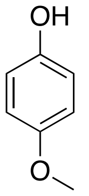

p-Cresol (3e). Performed according to general procedure 3. White solid (8 mg, 78%). Purified on silica with 10 % Et_2O in pentane (R_f = 0.15). **^{1}H NMR** (400 MHz, $CDCl_3$) δ 7.08 – 7.01 (m, 2H), 6.77 – 6.70 (m, 2H), 4.73 (s, 1H), 2.28 (s, 3H). **^{13}C{^{1}H} NMR** (101 MHz, $CDCl_3$) δ 153.2, 130.1, 130.0, 115.1, 20.5. **MS(EI)**: Calculated for $C_7H_8O^+$ 108.0570; found 108.1.

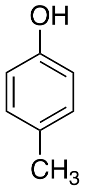

4-Chlorophenol (3f). Performed according to general procedure 3. White solid (12 mg, 93%). Purified on silica with 10 % Et_2O in pentane (R_f = 0.19). **^{1}H NMR** (400 MHz, $CDCl_3$) δ 7.23 – 7.15 (m, 2H), 6.81 – 6.72 (m, 2H), 4.90 (s, 1H). **^{13}C{^{1}H} NMR** (101 MHz, $CDCl_3$) δ 154.0, 129.5, 125.7, 116.6. **MS(EI)**: Calculated for $C_6H_5ClO^+$ 128.0023; found 128.0.

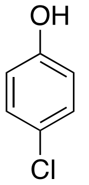

V. Oxidative fragmentation of ethers and acetals

4-bromo-1-phenylbutan-1-one (4d). Prepared according to general procedure 4. Colorless oil (20 mg, 89 %). Purified on silica with 2 % EtOAc in petroleum ether as eluent (R_f = 0.26). **^{1}H NMR** (400 MHz, $CDCl_3$) δ 8.02 – 7.95 (m, 2H), 7.61 – 7.53 (m, 1H), 7.51 – 7.46 (m, 2H), 3.56 (t, *J* = 6.3 Hz, 2H), 3.19 (t, *J* = 6.9 Hz, 2H), 2.32 (tt, *J* = 6.9, 6.3 Hz, 2H). **^{13}C{^{1}H} NMR** (101 MHz, $CDCl_3$) δ 199.0, 136.9, 133.4, 128.8, 128.2, 36.7, 33.8, 27.0. **MS(ESI)**: calculated for $C_{10}H_{12}BrO^+$ $[(M+H)^+]$ 227.0072; found 227.1.

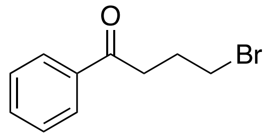

5-bromo-1-phenylpentan-1-one (4e). Prepared according to general procedure 4. Colorless oil (22 mg, 92 %). Purified on silica with 2 % EtOAc in petroleum ether as eluent (R_f = 0.19). **^{1}H NMR** (400 MHz, $CDCl_3$) δ 7.98 – 7.91 (m, 2H), 7.61 – 7.54 (m, 1H), 7.52 – 7.43 (m, 2H), 3.46 (t, *J* = 6.4 Hz, 2H), 3.02 (t, *J* = 6.9 Hz, 2H), 2.02 – 1.85 (m, 4H). **^{13}C{^{1}H} NMR** (101 MHz, $CDCl_3$) δ 199.7, 137.0, 133.2, 128.8, 128.1, 37.5, 33.5, 32.3, 22.9. **MS(ESI)**: calculated for $C_{11}H_{14}BrO^+$ $[(M+H)^+]$ 241.0228; found 241.0.

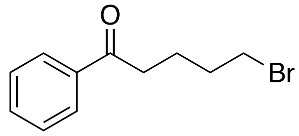

Methyl benzoate (4f). Prepared according to general procedure 4. Colorless oil (16 mg, 99 %). Filtered through a short silica plug eluted with DCM. **^{1}H NMR** (400 MHz, $CDCl_3$) δ 8.08 – 8.01 (m, 2H), 7.59 – 7.53 (m, 1H), 7.48 – 7.42 (m, 2H), 3.92 (s, 3H). **^{13}C{^{1}H} NMR** (101 MHz, $CDCl_3$) δ 167.3, 133.0, 130.3, 129.7, 128.5, 52.2. **MS(EI)**: calculated for $C_8H_8O_2^+$ 136.0524; found 136.1.

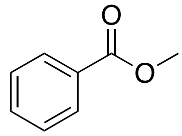

VI. Alkene isomerization

(Z)-ethyl cinnamate (5d). Prepared according to general procedure 5. Colorless oil (10 mg, 56%). Purified on preparatory TLC with 2 % Et_2O in pentane (R_f = 0.14). **^{1}H NMR** (400 MHz, $CDCl_3$) δ 7.62 – 7.51 (m, 2H), 7.42 – 7.29 (m, 3H), 6.96 (d, *J* = 0.9 Hz, 1H), 5.96 (d, *J* = 12.6 Hz, 1H), 4.18 (q, *J* = 7.1 Hz, 2H), 1.25 (t, *J* = 7.1 Hz, 3H). **^{13}C{^{1}H} NMR** (101 MHz, $CDCl_3$) δ 166.2, 143.0, 134.9, 129.7, 129.0, 128.0, 119.9, 60.3, 14.1. **MS(EI)**: calculated for $C_{11}H_{12}O_2^+$ 176.0832; found 176.1.

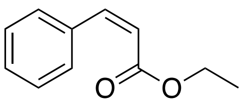

(Z)-cinnamaldehyde (5e). Prepared according to general procedure 5. Colorless oil (12 mg, 92%). Purified on silica with 2 % EtOAc in hexane (R_f = 0.07). **^{1}H NMR** (400 MHz, $CDCl_3$) δ 9.97 (d, *J* = 8.2, 1H), 7.63 (d, *J* = 11.6, 1H), 7.46 – 7.37 (m, 5H), 6.25 – 6.10 (m, 1H). **^{13}C{^{1}H} NMR** (101 MHz, $CDCl_3$) δ 192.6, 148.7, 134.2, 130.4, 129.8, 129.8, 128.6. **MS(EI)**: calculated for $C_9H_8O^+$ 132.0570; found 132.1.

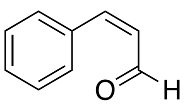

2H-chromen-2-one (5f). Prepared according to general procedure 5. White solid (14 mg, 94%). Filtered over a short silica plug eluted with 20 % EtOAc in pentane (R_f = 0.22). **^{1}H NMR** (400 MHz, $CDCl_3$) δ 7.71 (d, *J* = 9.5 Hz, 1H), 7.57 – 7.45 (m, 2H), 7.36 – 7.24 (m, 2H), 6.42 (d, *J* = 9.6 Hz, 1H). **^{13}C{^{1}H} NMR** (101 MHz, $CDCl_3$) δ 160.8, 154.0, 143.5, 131.8, 127.9, 124.4, 118.8, 116.9, 116.7. **MS(EI)**: $C_9H_6O_2^+$ 146.0368; found 146.0.

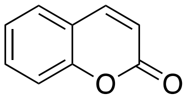

UV-Vis Spectroscopy (solvent stability)

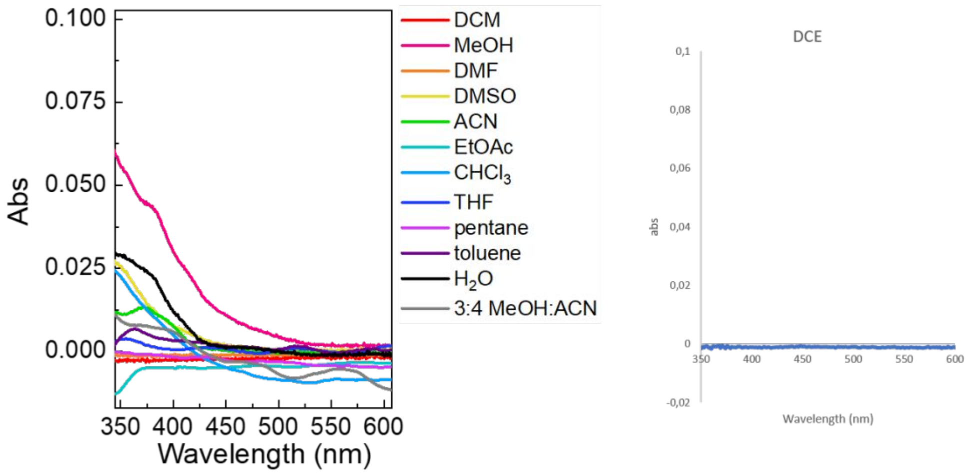

Figure S1. UV-Vis spectra of solutions after solvent stability tests. During the tests, Al_2O_3-Ir was stirred (1000 RPM) in 2.0 ml of solvent under irradiation with a blue Kessil LED lamp for two hours; 3:4 MeOH:ACN was stirred for 30 minutes. The catalyst was filtered off and UV-Vis spectra recorded. Ir(dcabpy)(ppy)$_2$ desorption is seen in acetonitrile, methanol, water, chloroform, and dimethyl sulfoxide due to the appearance of the broad band at 370 nm characteristic of Ir(dcabpy)(ppy)$_2$.

Reaction rate comparison of homogeneous vs heterogenized catalysts

Table S1. Reaction yields over time for the heterogenized Al_2O_3-Ir catalyst to the homogeneous Ir(dcabpy)(ppy)$_2$. Reaction yields as a function of time for the dehalogenation of 2-bromoacetophenone to acetophenone (left), oxidative hydroxylation of 4-chlorophenylboronic acid to 4-chlorophenol (middle), and isomerization of (E)-ethyl cinnamate to (Z)-ethyl cinnamate (right).

**Reductive Dehalogenation**			**Oxidative Hydroxylation**			**Isomerization**		
**Time (min)**	**Yield (%) Al_2O_3-Ir**	**Yield (%) Ir homog.**	**Time (h)**	**Yield (%) Al_2O_3-Ir**	**Yield (%) Ir homog**	**Time (h)**	**Yield (%) Al_2O_3-Ir**	**Yield (%) Ir homog**
**7**	24	0	**1**	19	13	**1**	3	0
**15**	49	20	**3**	40	26	**3**	6	1
**30**	52	30	**5**	55	37	**5**	11	2
**60**	77	57	**20**	82	64	**20**	33	13
**120**	96	82	-	-	-	-	-	-

Reusability Studies

Table S2. Results of catalyst reusability tests. Shown are the normalized reaction yields for dehalogenation of 2-bromoacetophenone to acetophenone, oxidative hydroxylation of 4-chlorophenylboronic acid to 4-chlorophenol, and isomerization of (E)-ethyl cinnamate to (Z)-ethyl cinnamate. Catalysts were reused 3-5 times. After each use, the catalyst was centrifuged from the reaction mixture, rinsed with reaction solvent, and dried under vacuum for an hour. Once dry, the catalyst was subjected to the same reaction again.

**Reaction Number**	**Normalized Yield (%): Dehalogenation**	**Normalized Yield (%): Oxidative Hydroxylation**	**Normalized Yield (%): Isomerization**
**1**	100	100	100
**2**	95	62	50
**3**	71	50	25
**4**	79	-	-
**5**	62	-	-

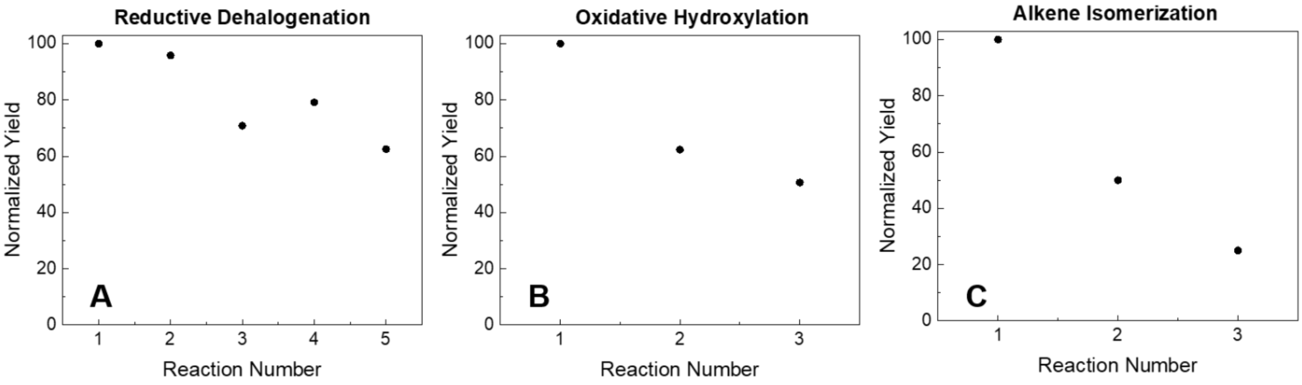

Figure S2. Catalyst reusability results for (A) reductive dehalogenation of 2-bromoacetophenone to acetophenone (B) oxidative hydroxylation of 4-chlorophenylboronic acid to 4-chlorophenol and (C) isomerization of (E)-ethyl cinnamate to (Z)-ethyl cinnamate. Reductive dehalogenation reactions were repeated five times with the same catalyst, while the oxidative hydroxylation and isomerization reactions were repeated three times. Normalized yields are plotted to highlight how much the yield changed upon reusing the catalyst for each subsequent reaction.

^{1}H and ^{13}C NMR Spectra

Acetophenone (1e), (400 MHz, CDCl3)

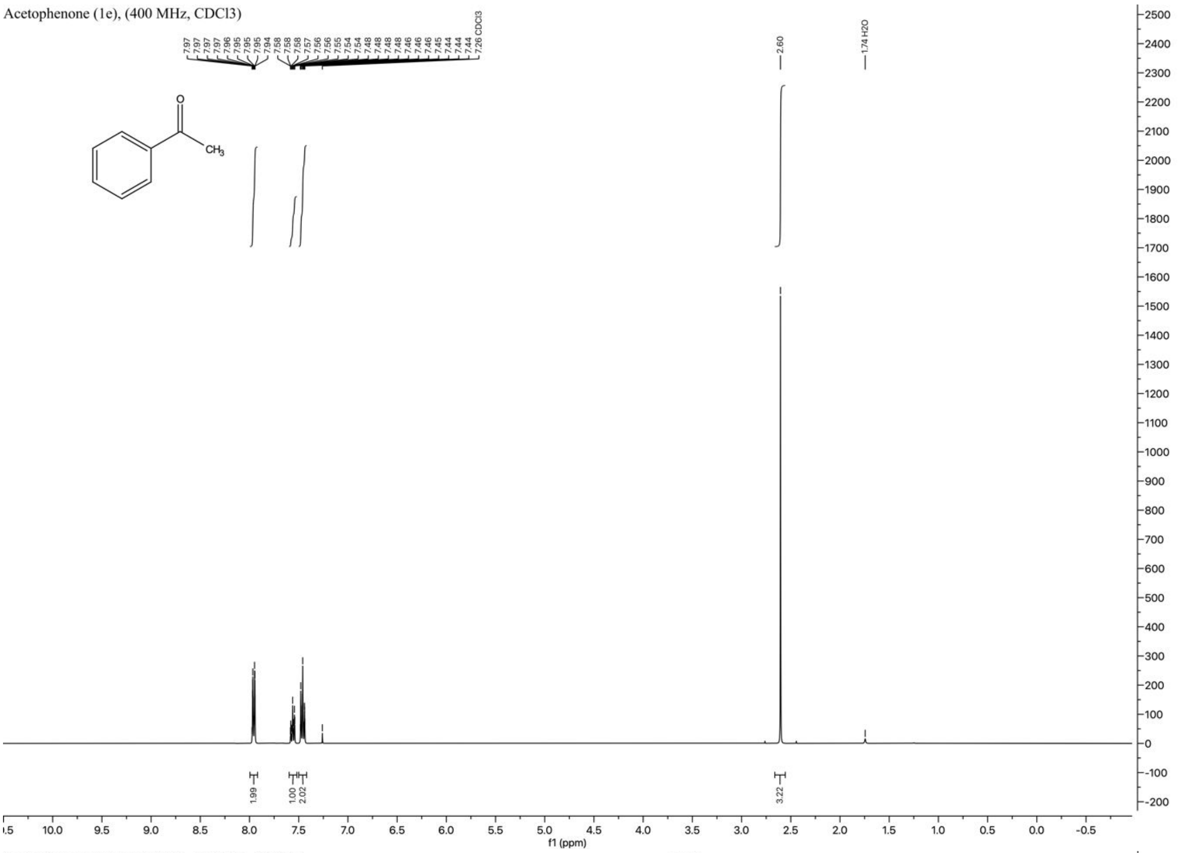

Acetophenone (1e), (13C{1H} 101 MHz, CDCl3)

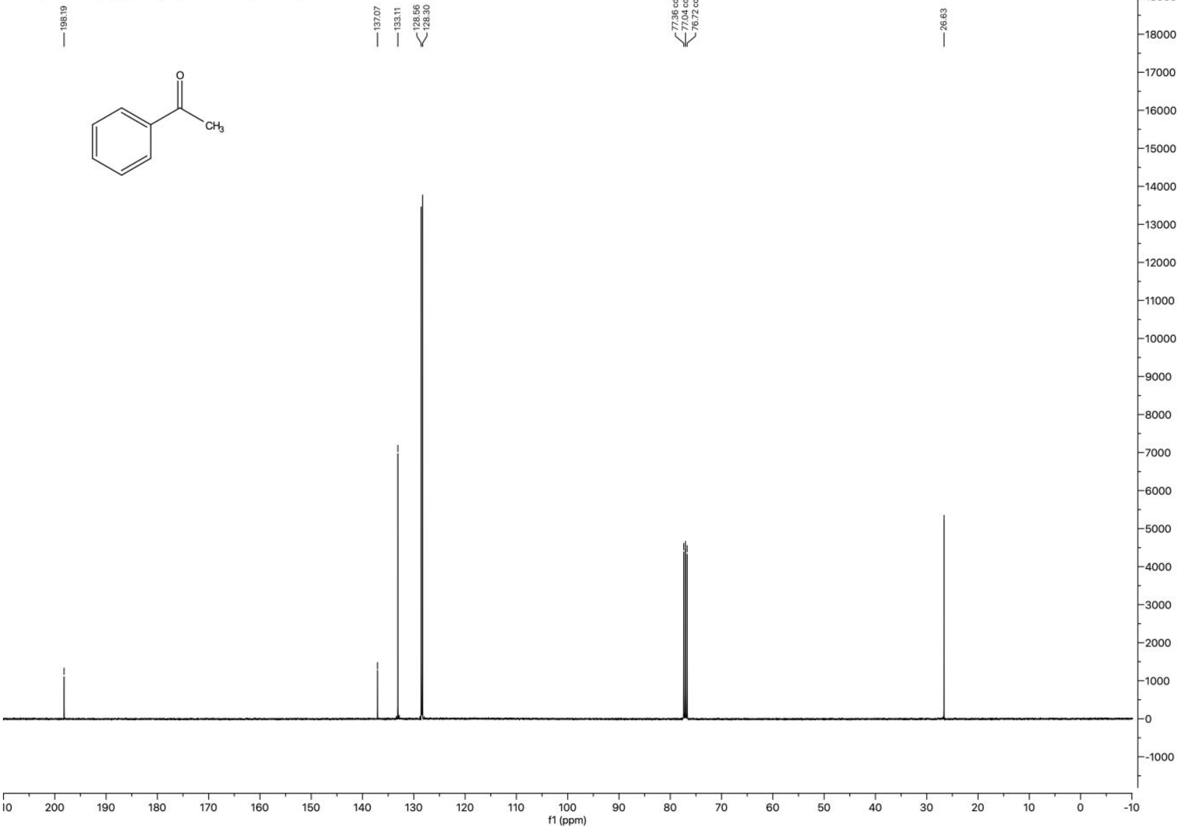

Bibenzyl (1f), (400 MHz, CDCl3)

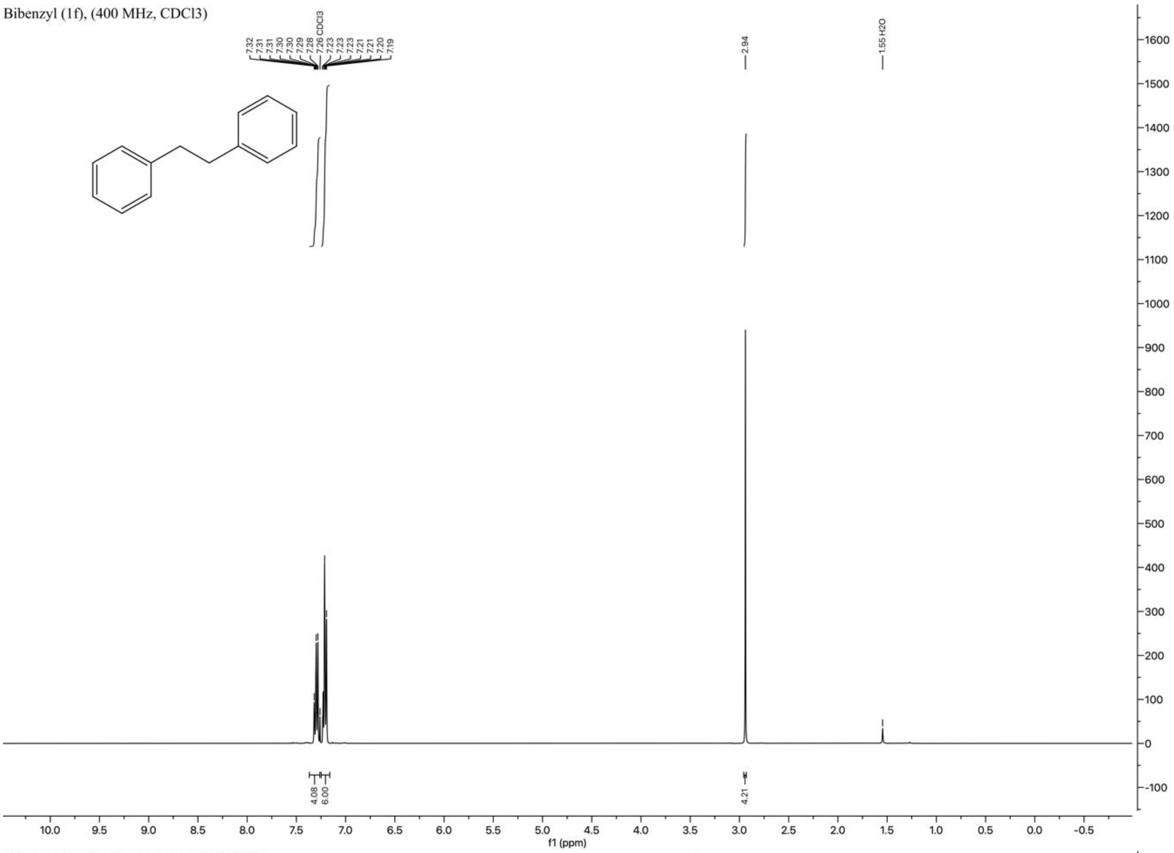

Bibenzyl (1f), (13C{1H} 101 MHz, CDCl3)

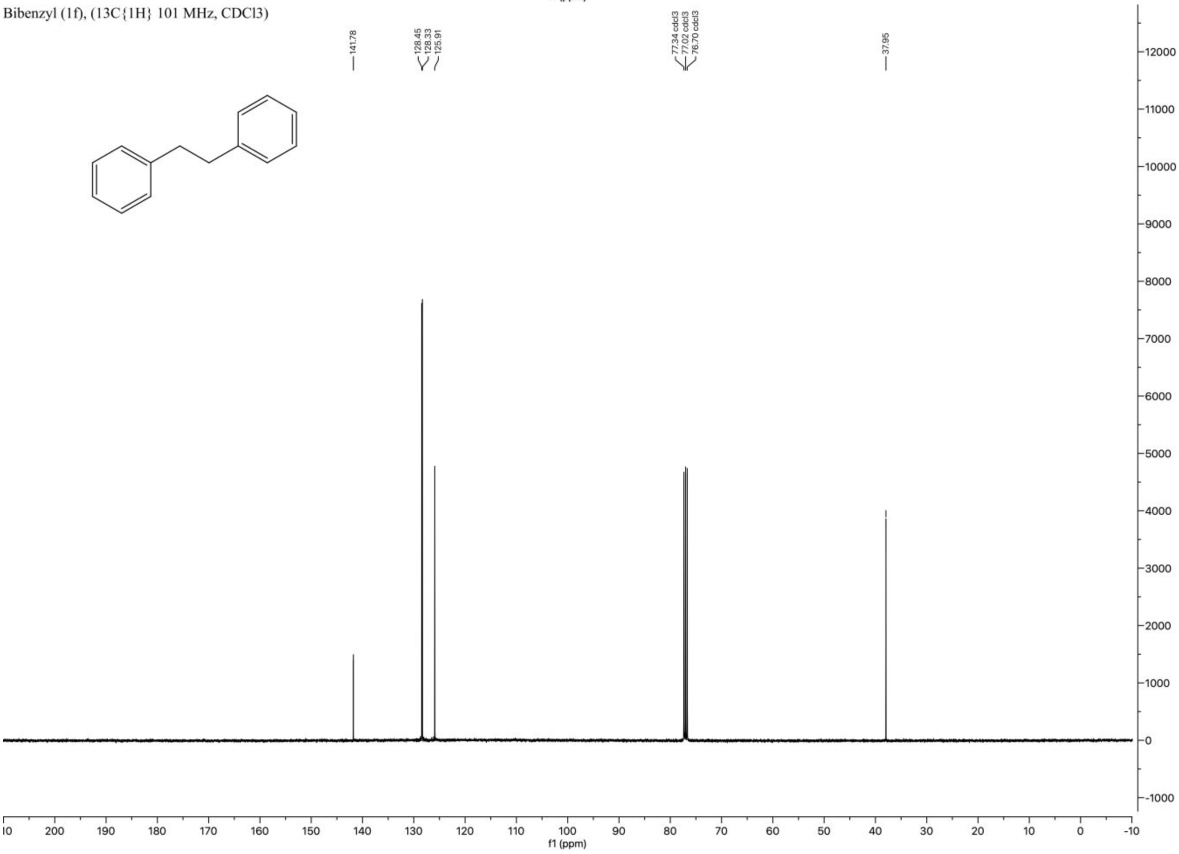

Diethyl malonate (1g), (400 MHz, CDCl3)

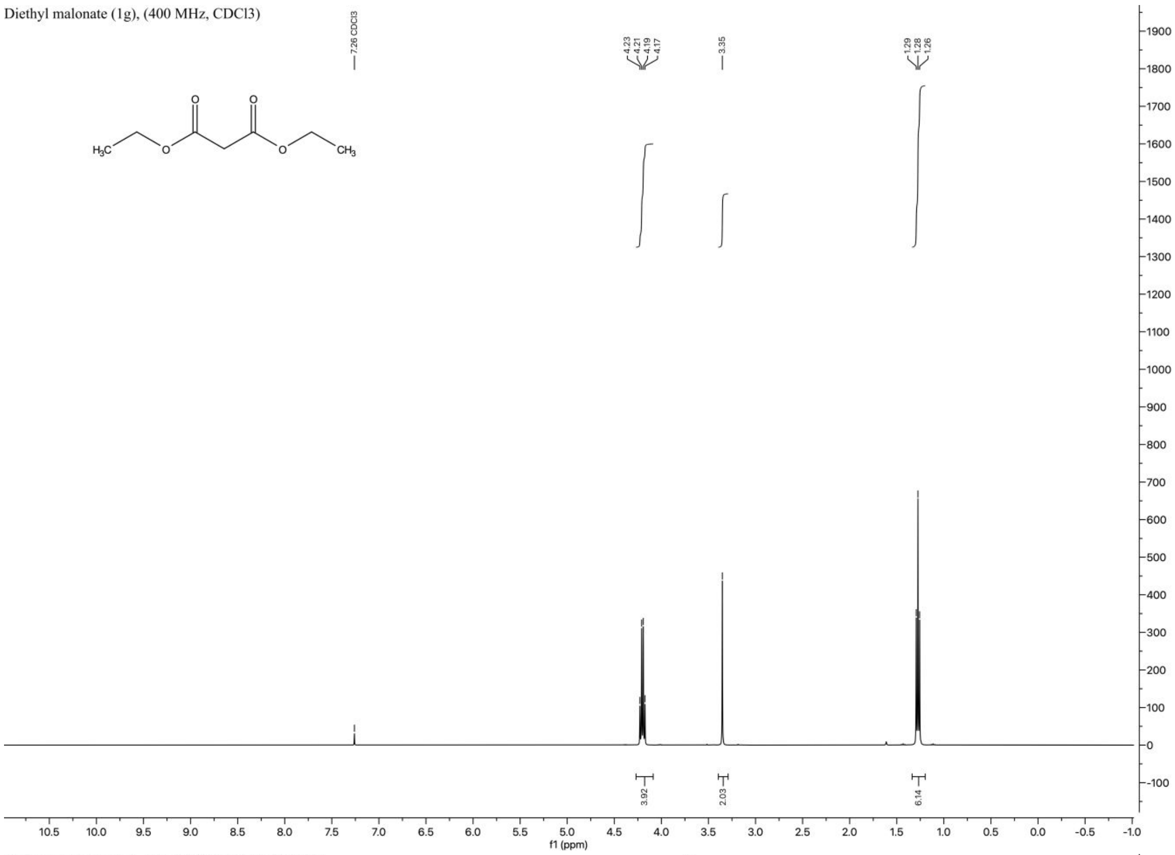

Diethyl malonate (1g), (13C{1H} 400 MHz, CDCl3)

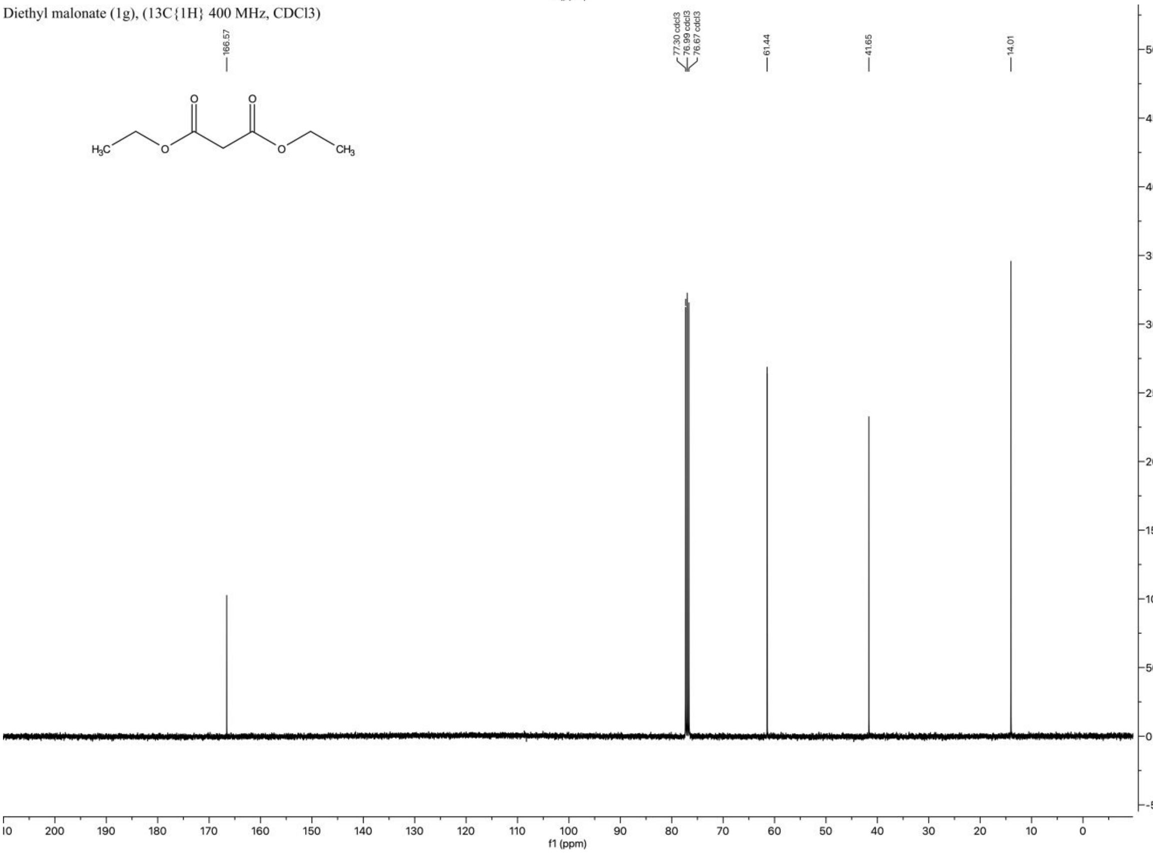

Diethyl 2-methylmalonate (1h), (400 MHz, CDCl3)

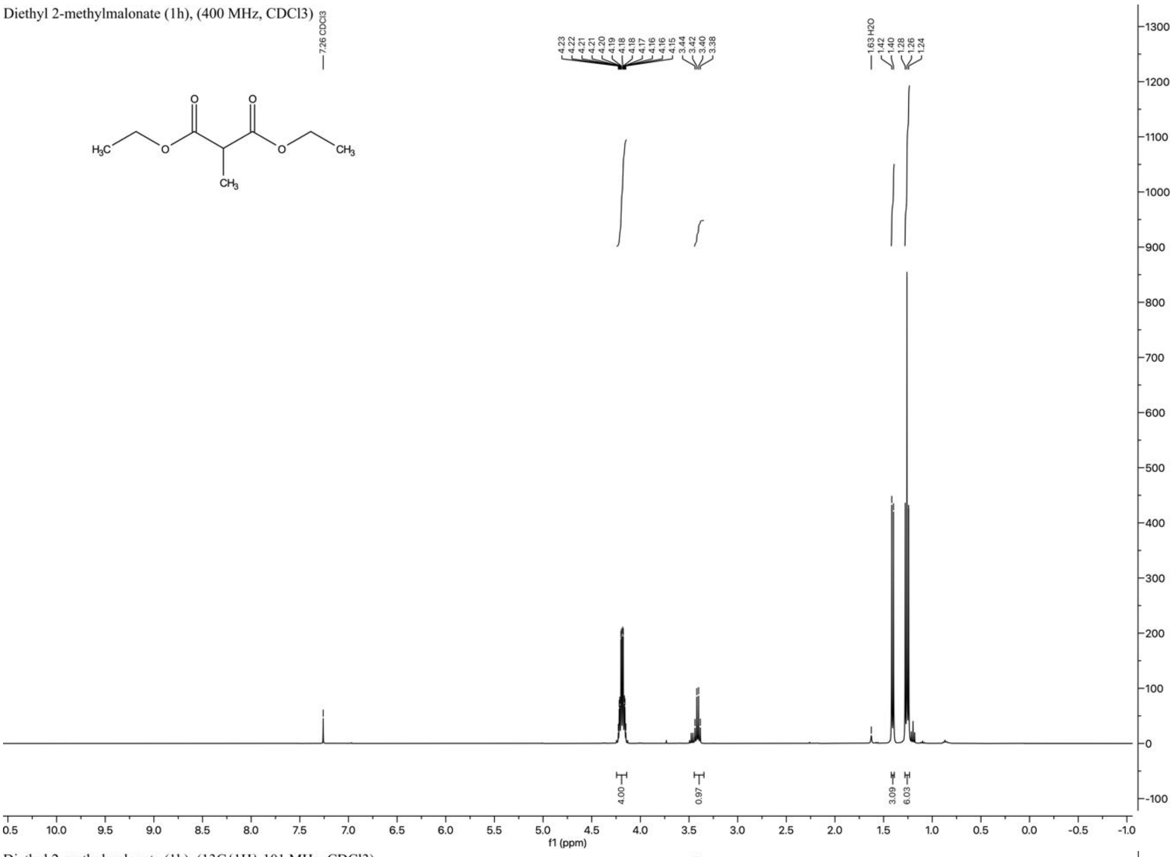

Diethyl 2-methylmalonate (1h), (13C{1H} 101 MHz, CDCl3)

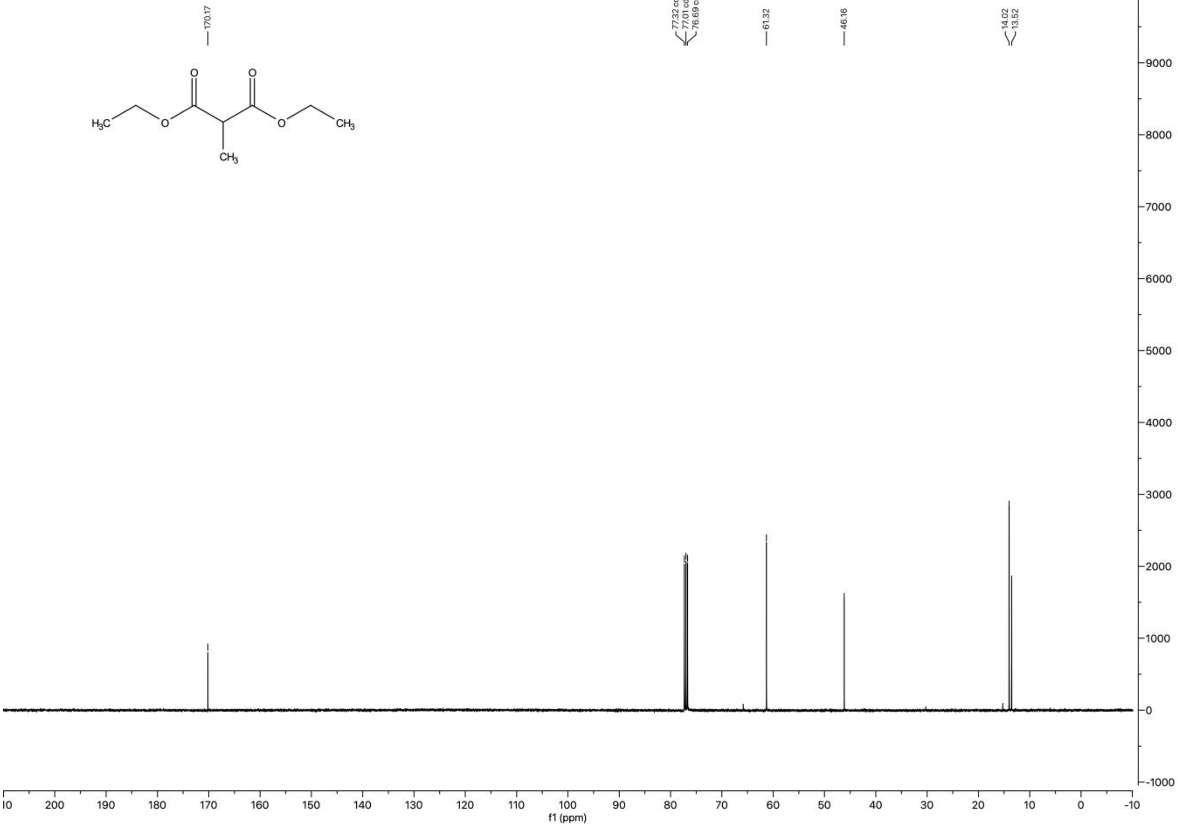

7,7,8,8,9,9,10,10,11,11,12,12,13,13,14,14,14-heptadecafluoro-5-iodotetradecan-1-ol (2c), (400 MHz, CDCl3)

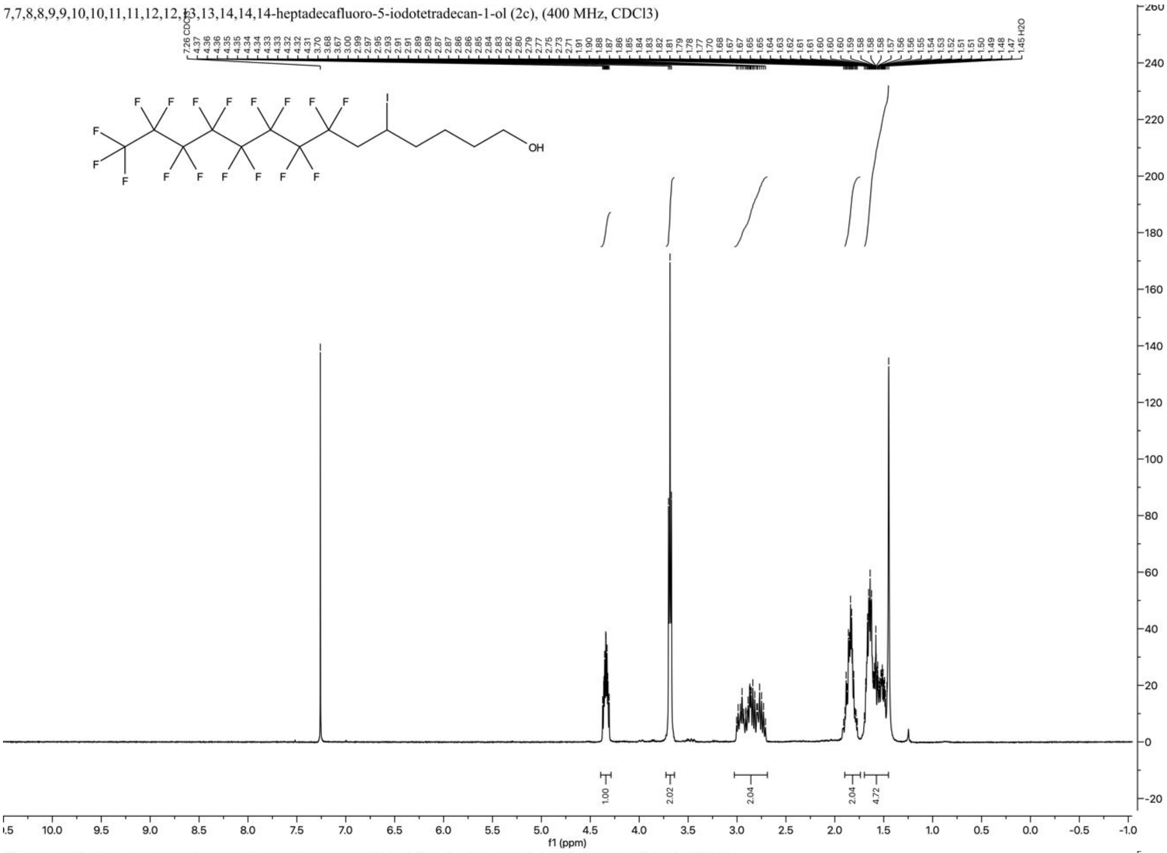

7,7,8,8,9,9,10,10,11,11,12,12,13,13,14,14,14-heptadecafluoro-5-iodotetradecan-1-ol (2c), (13C{1H} 101 MHz, CDCl3)

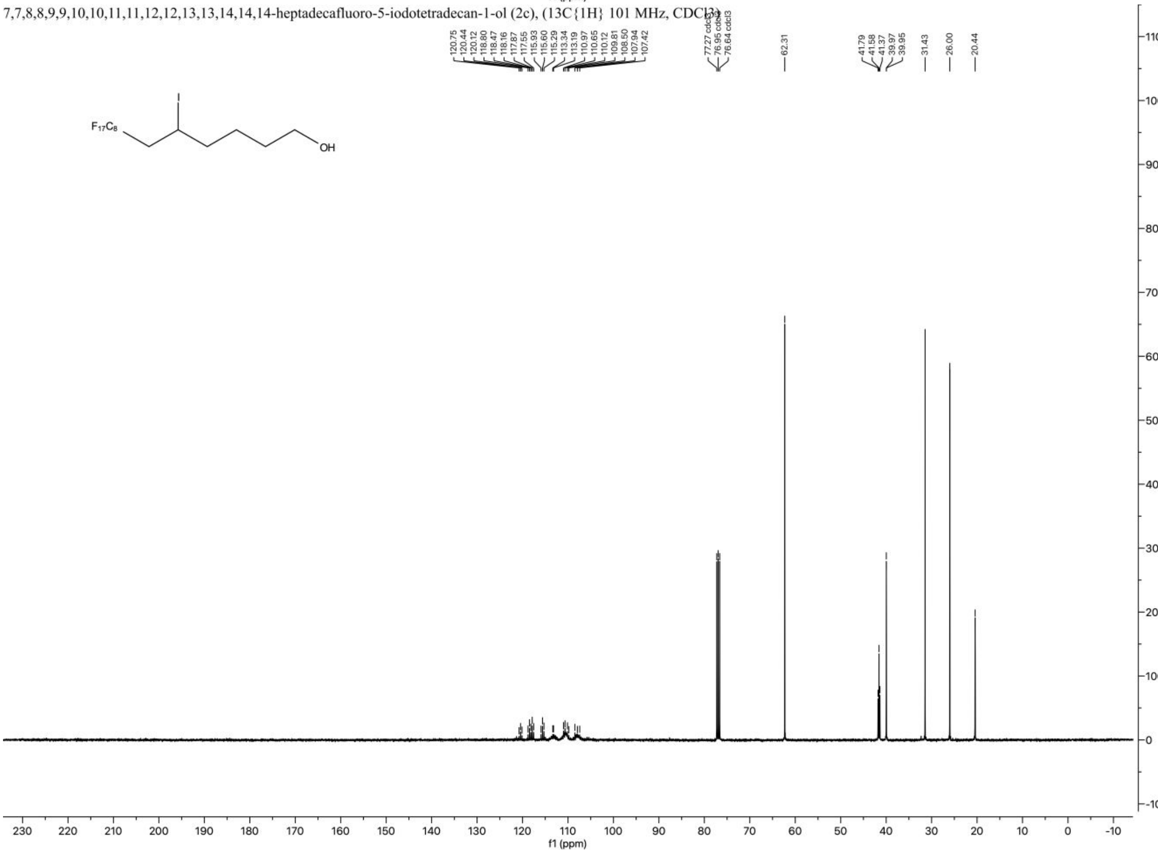

5-bromo-7,7,7-trichloroheptane-1-ol (2d), (400 MHz, CDCl3)

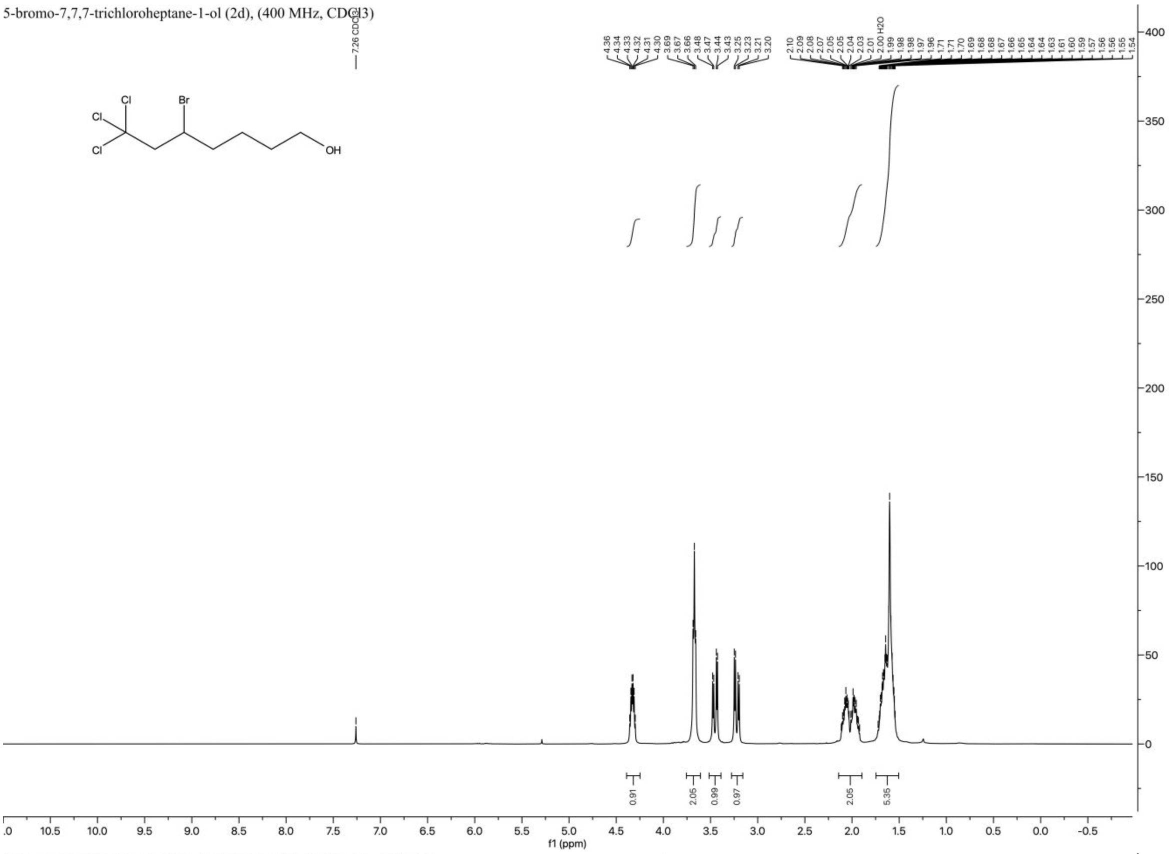

5-bromo-7,7,7-trichloroheptane-1-ol (2d), (13C{1H) 101 MHz, CDCl3)

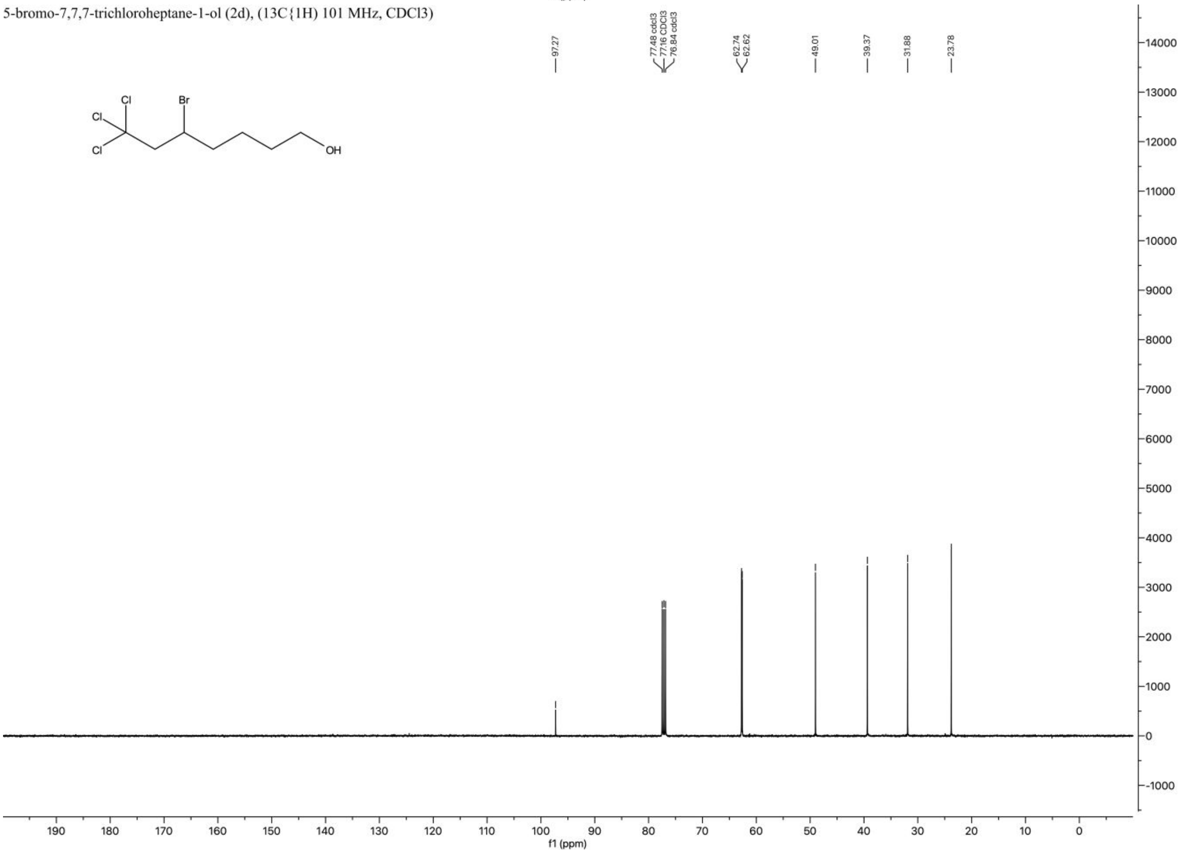

4-(2-bromopropan-2-yl)-1-(2,2,2-trichloroethyl)cyclohex-1-ene (2e), (400 MHz, CDCl3)

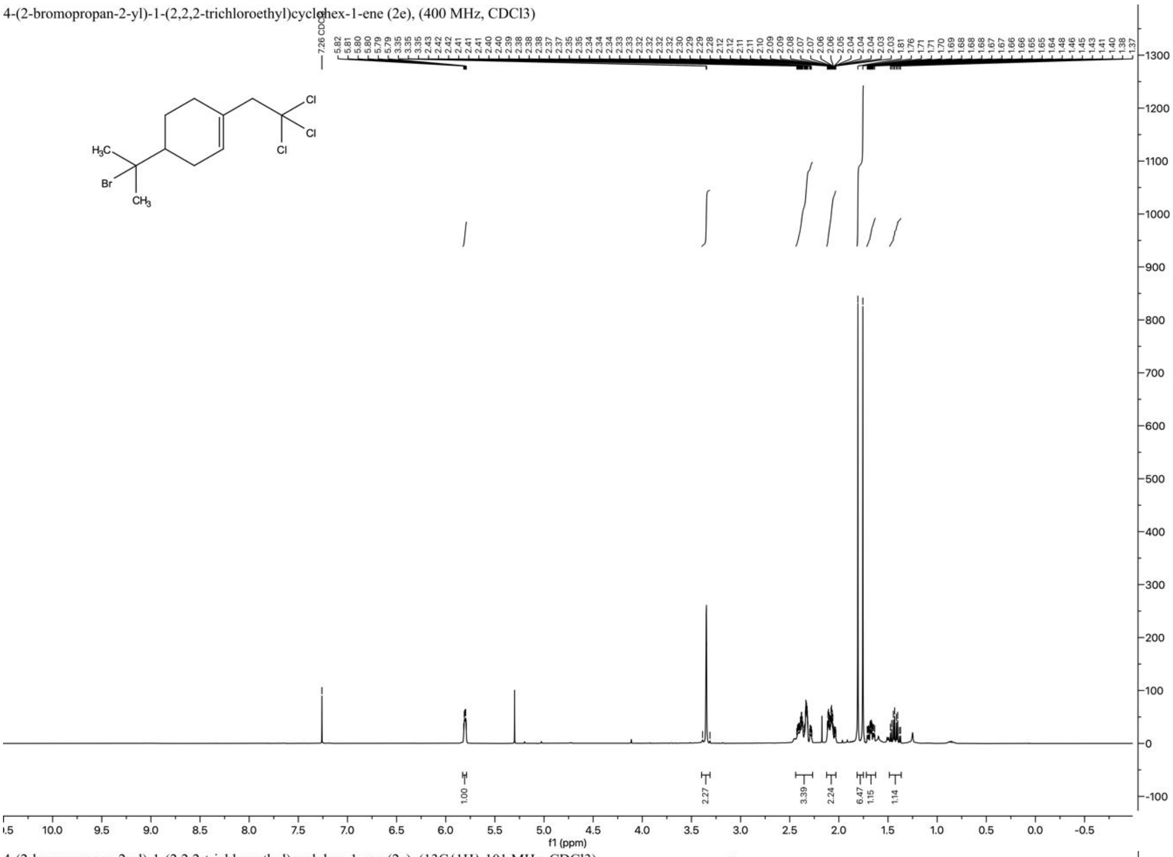

4-(2-bromopropan-2-yl)-1-(2,2,2-trichloroethyl)cyclohex-1-ene (2e), (13C{1H} 101 MHz, CDCl3)

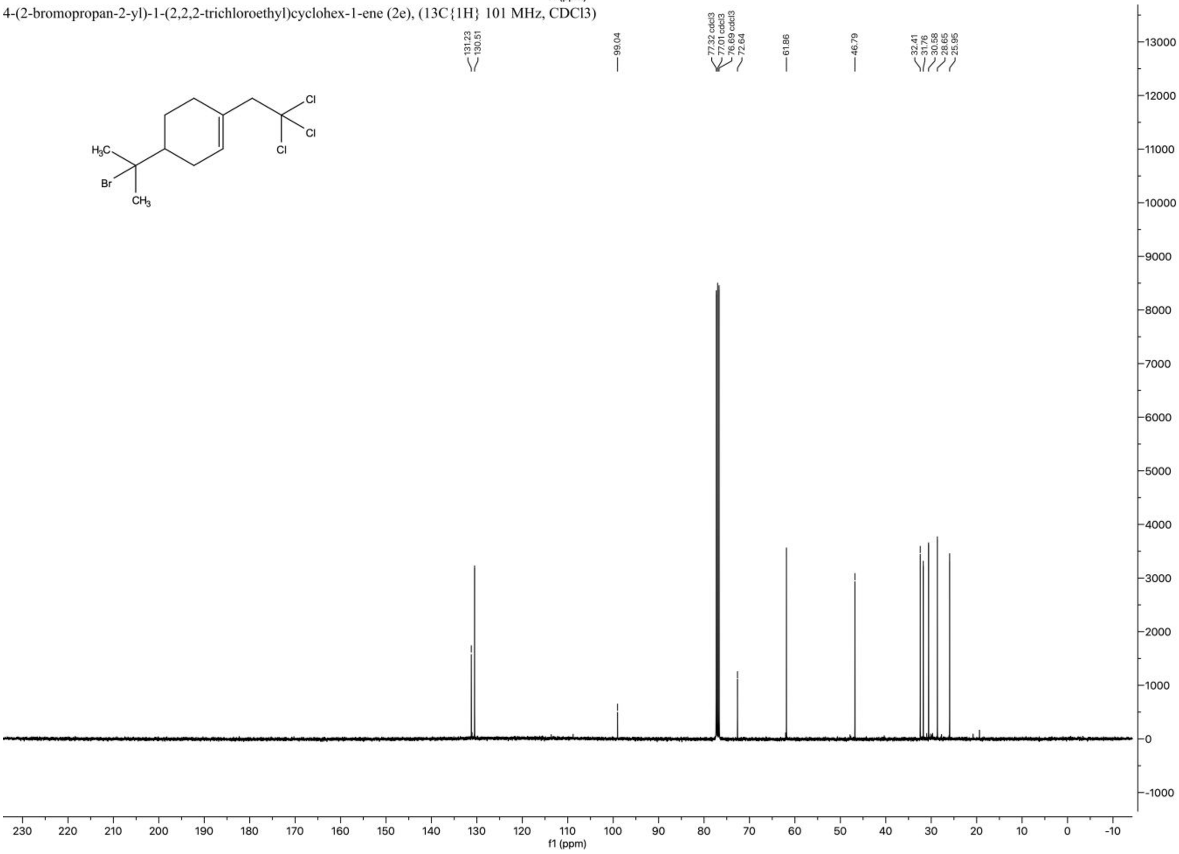

4-methoxyphenol (3d), (400 MHz, CDCl3)

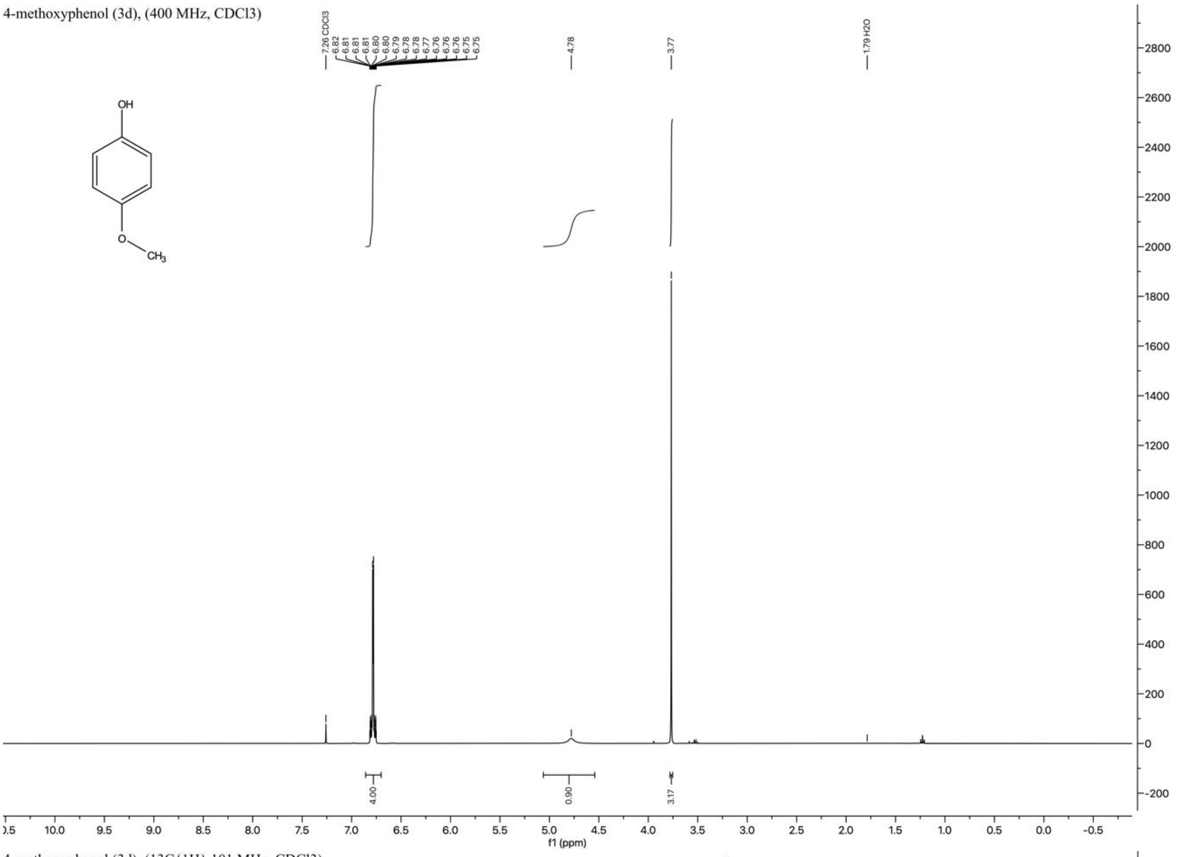

4-methoxyphenol (3d), (13C{1H} 101 MHz, CDCl3)

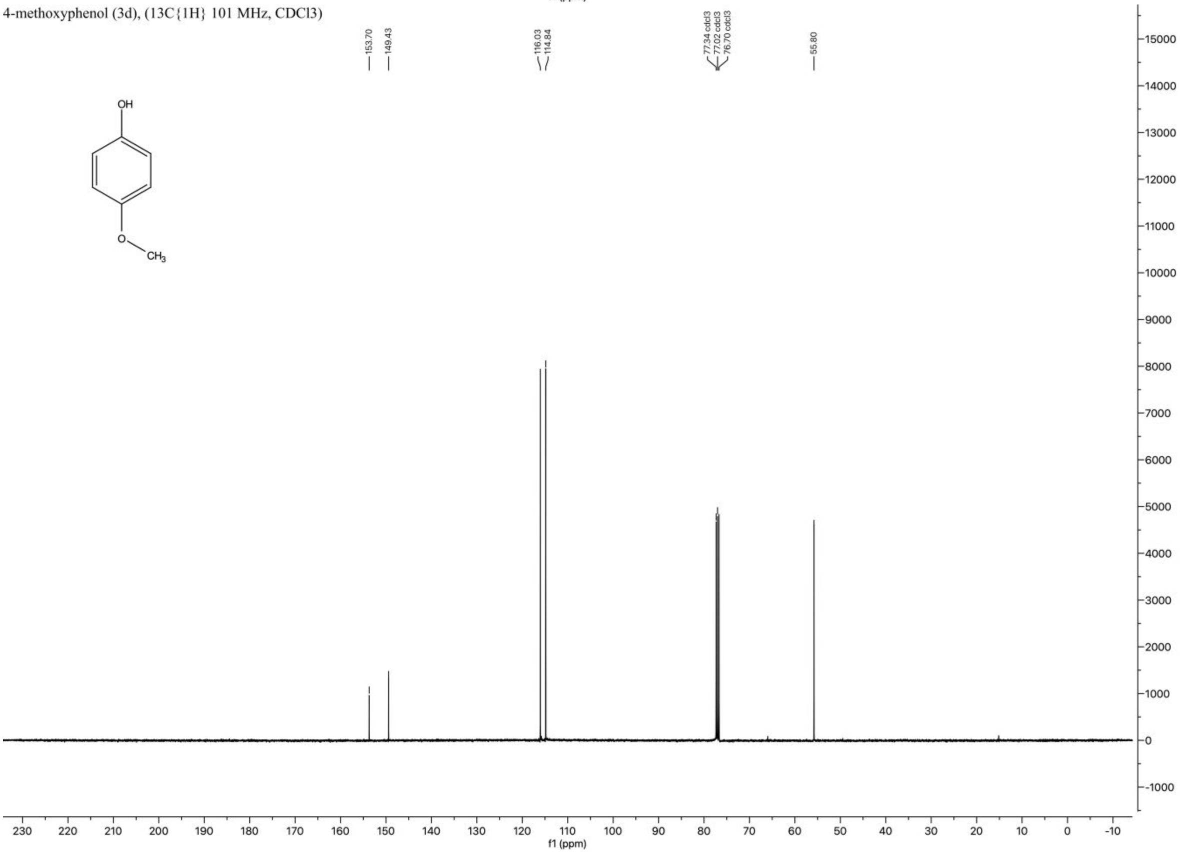

p-cresol (3e), (400 MHz, CDCl3)

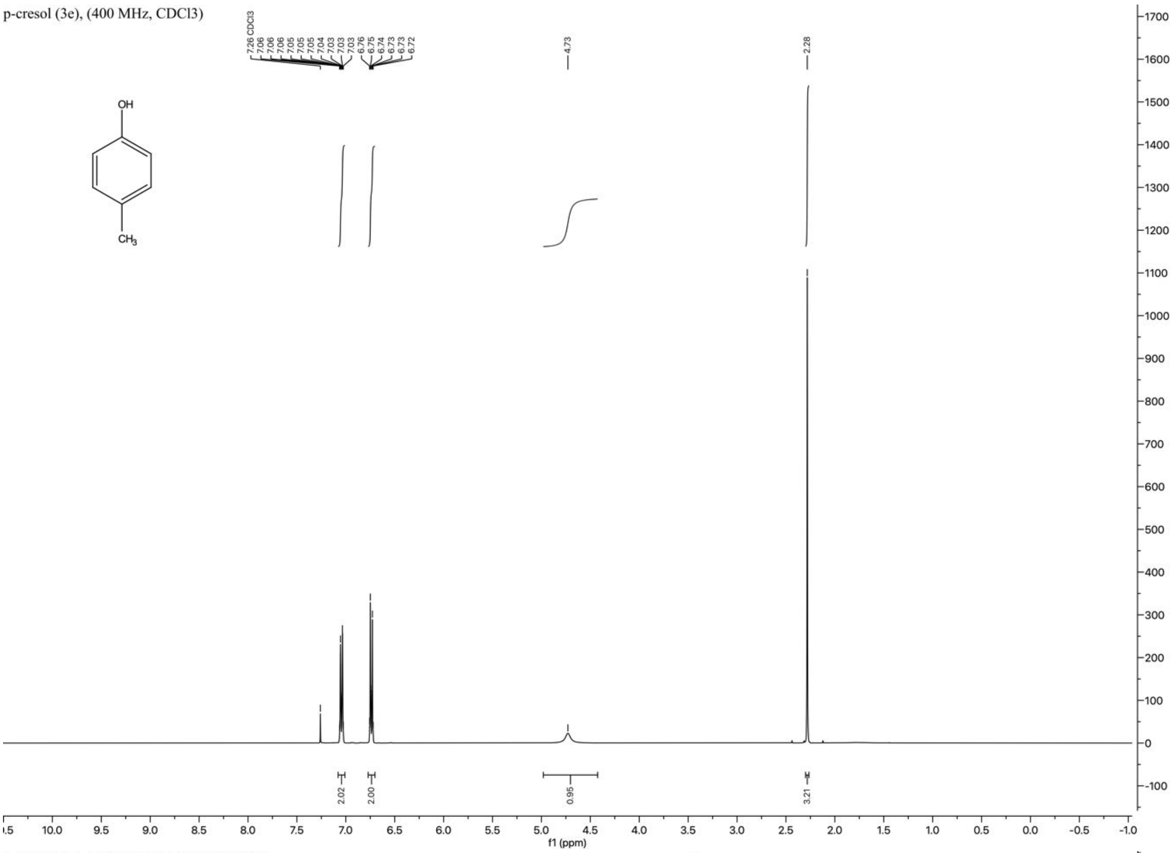

p-cresol (3e), (13C{1H} 101 MHz, CDCl3)

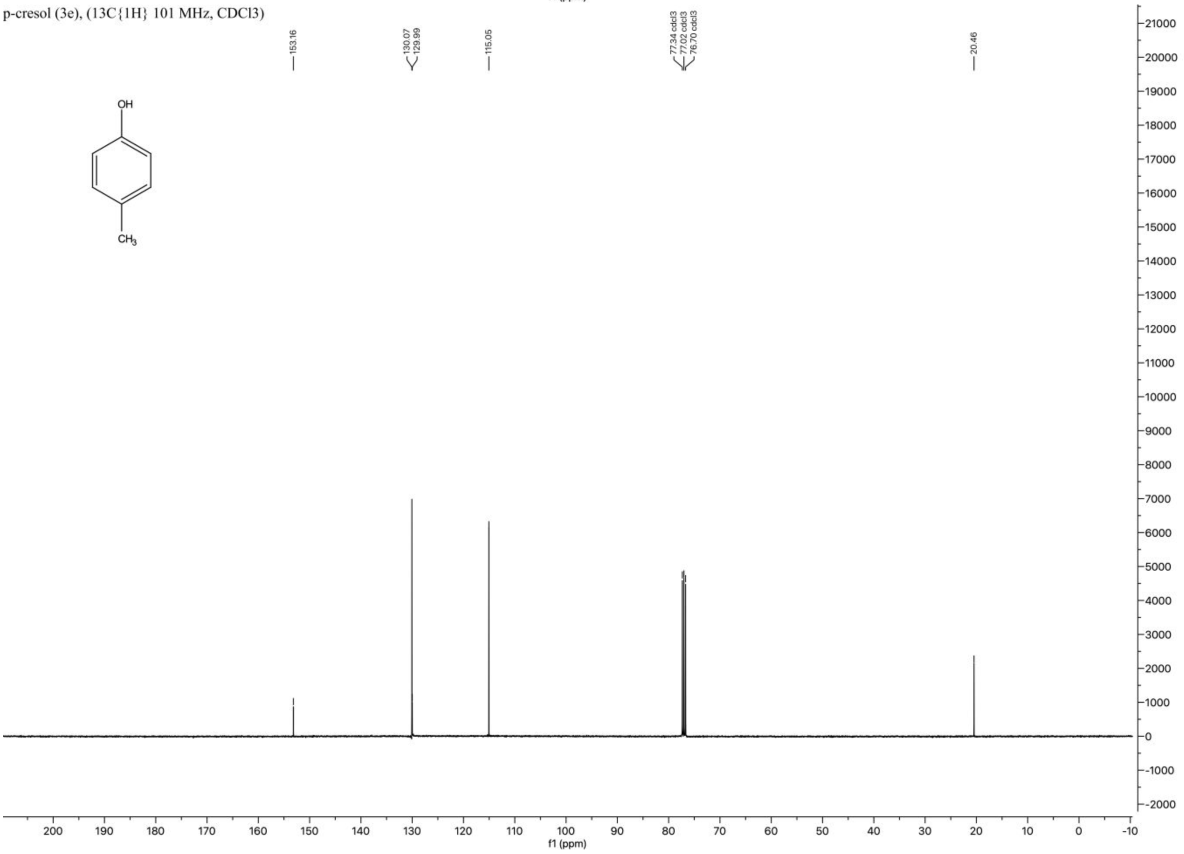

4-Chlorophenol (3f), (400 MHz, CDCl3)

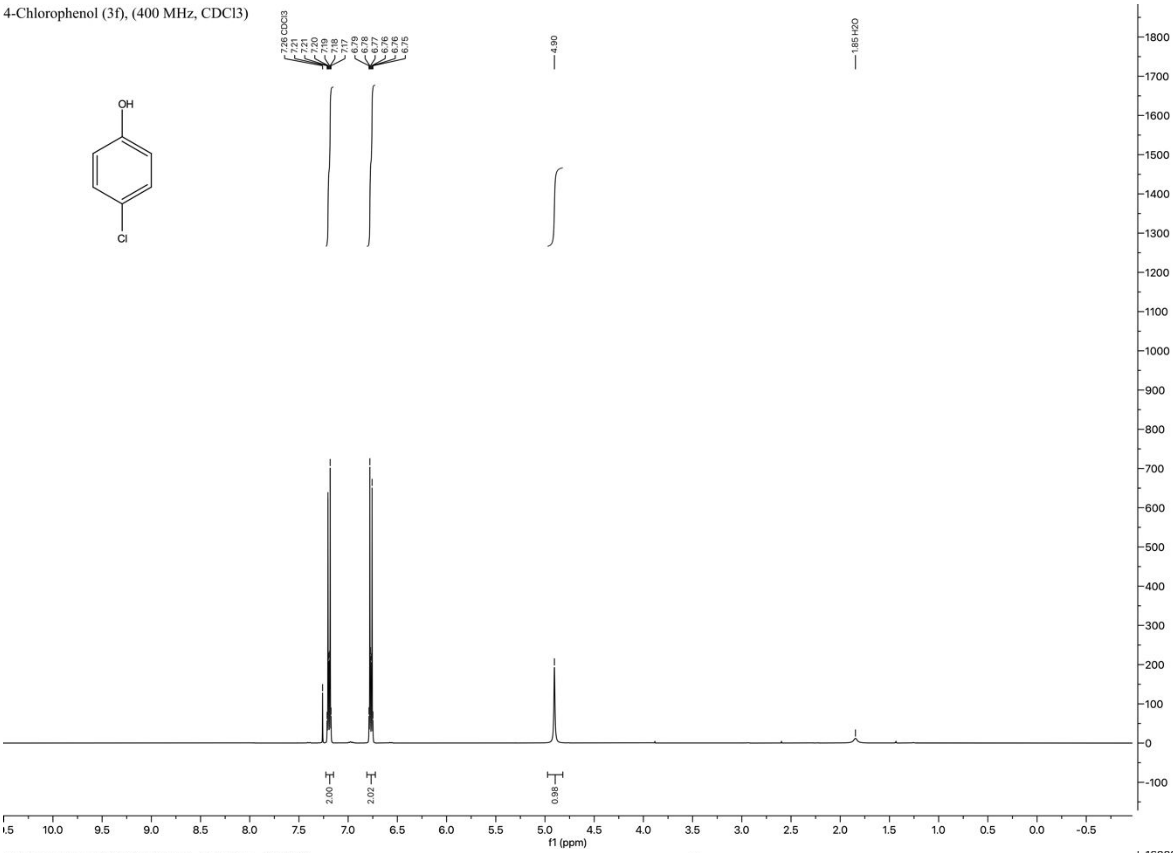

4-Chlorophenol (3f), (13C{1H} 101 MHz, CDCl3)

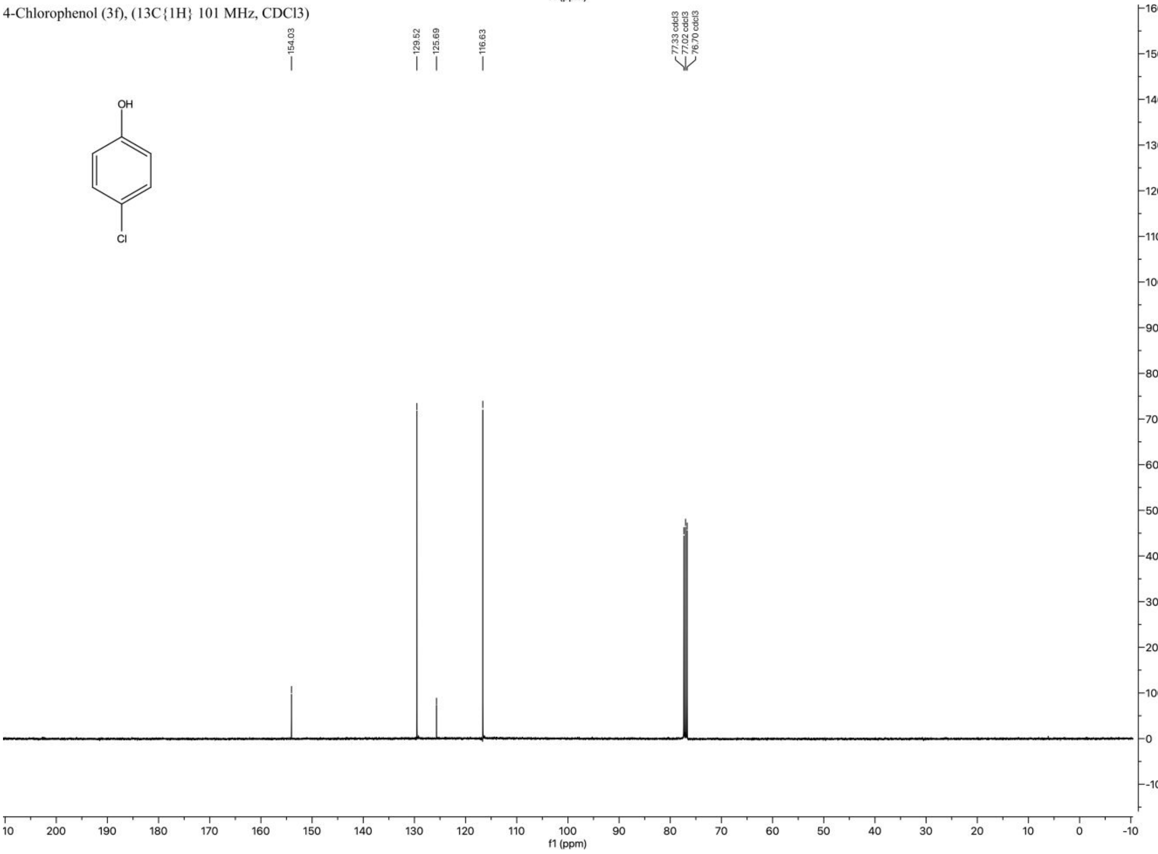

4-bromo-1-phenylbutan-1-one (4d), (400 MHz, CDCl3)

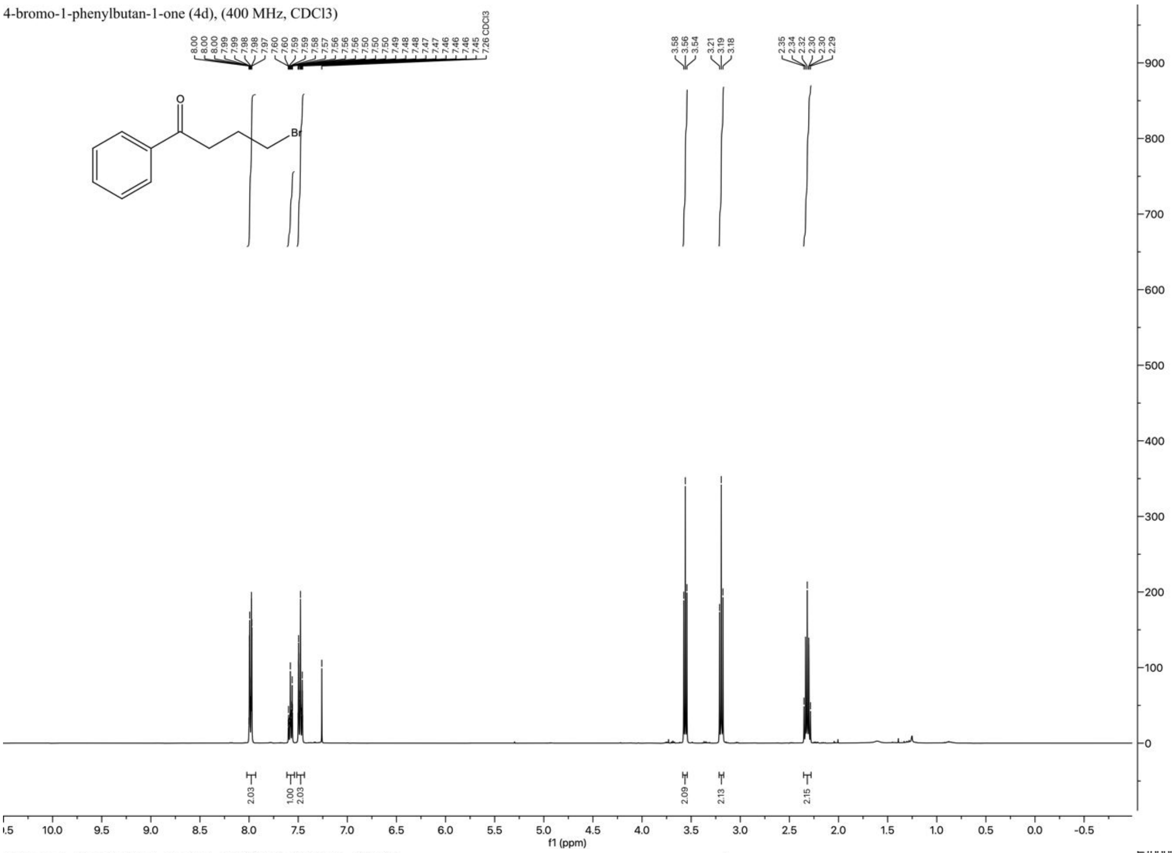

4-bromo-1-phenylbutan-1-one (4d), (13C{1H} 101 MHz, CDCl3)

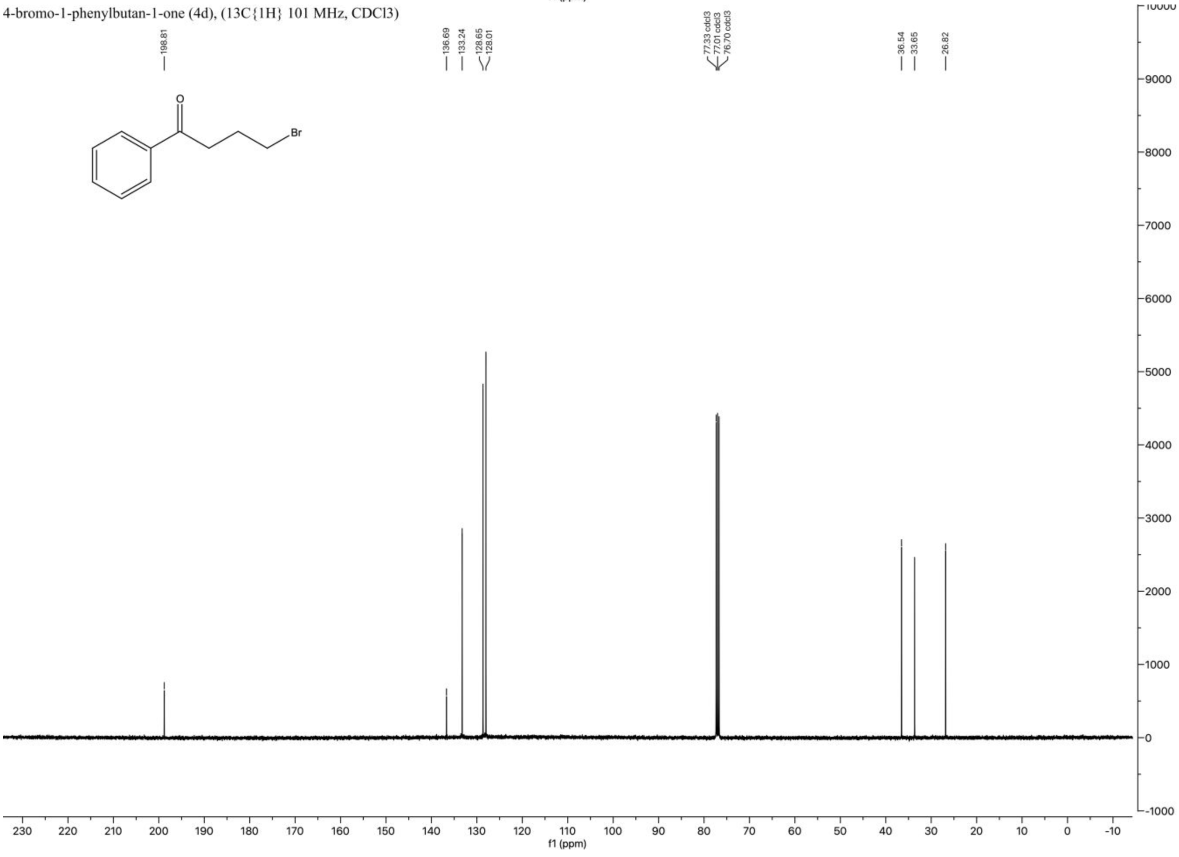

5-bromo-1-phenylpentan-1-one (4e), (400 MHz, CDCl3)

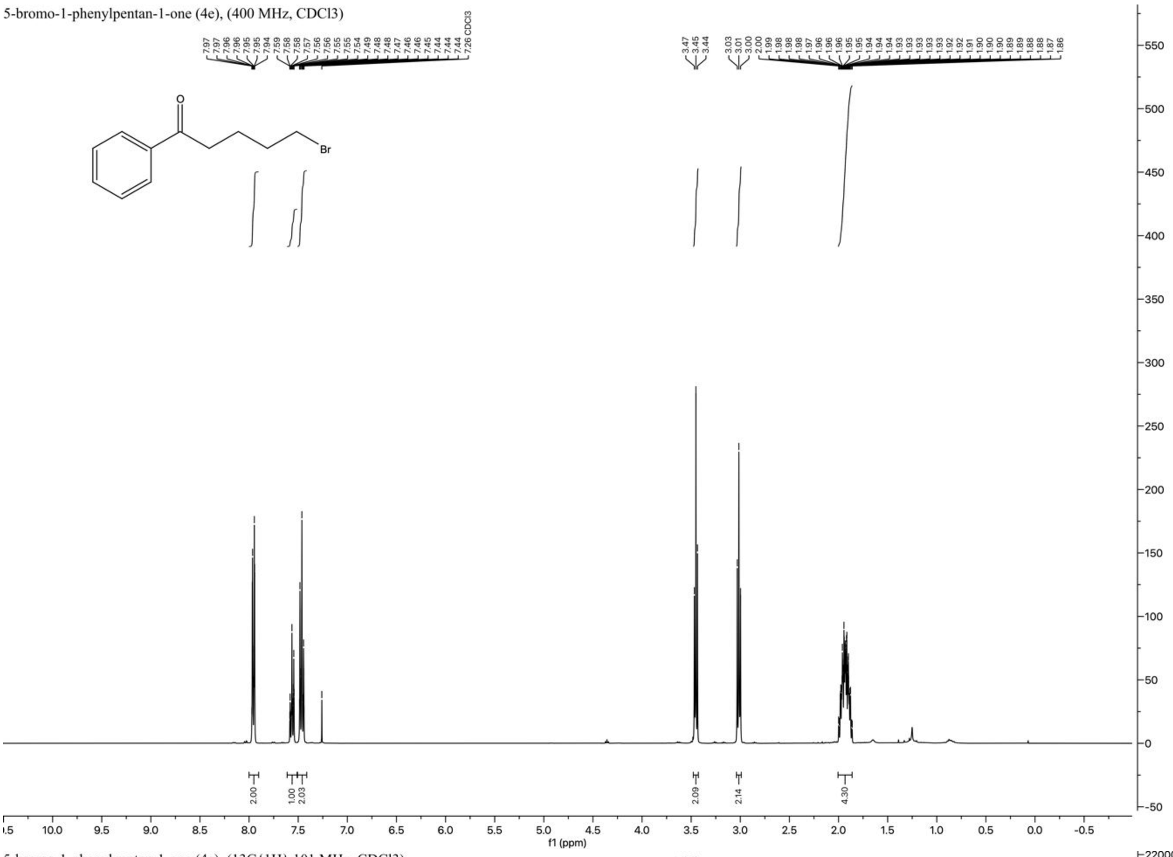

5-bromo-1-phenylpentan-1-one (4e), (13C{1H} 101 MHz, CDCl3)

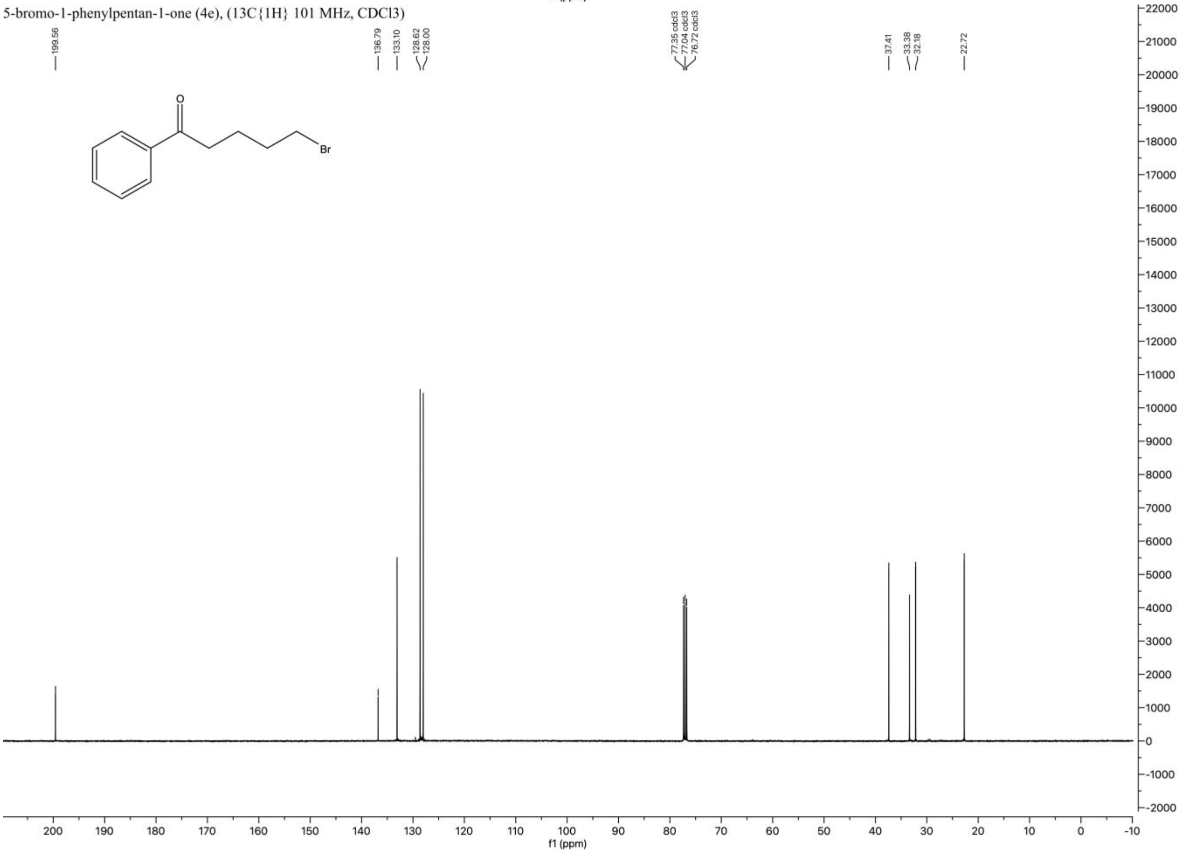

Methyl benzoate (4f), (400 MHz, CDCl3)

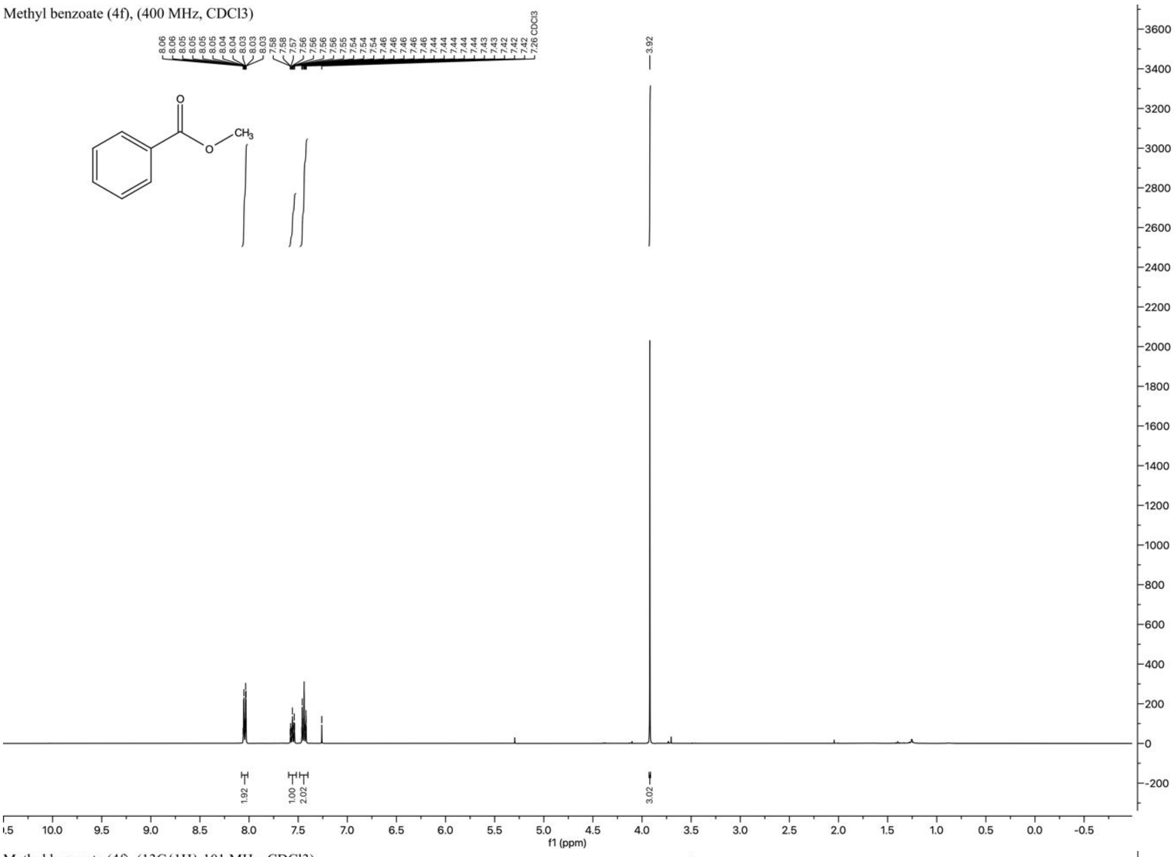

Methyl benzoate (4f), (13C{1H} 101 MHz, CDCl3)

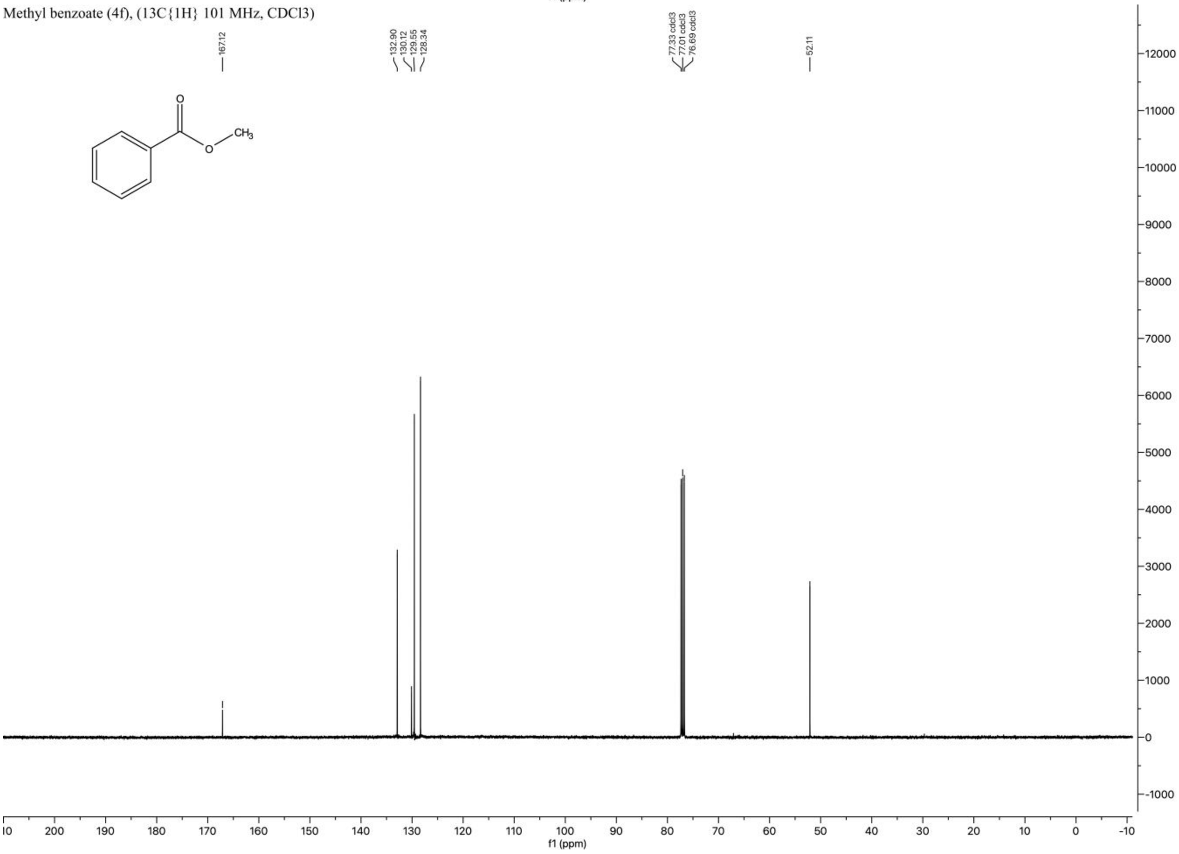

(Z)-ethyl cinnamate (5d), (400 MHz, CDCl3)

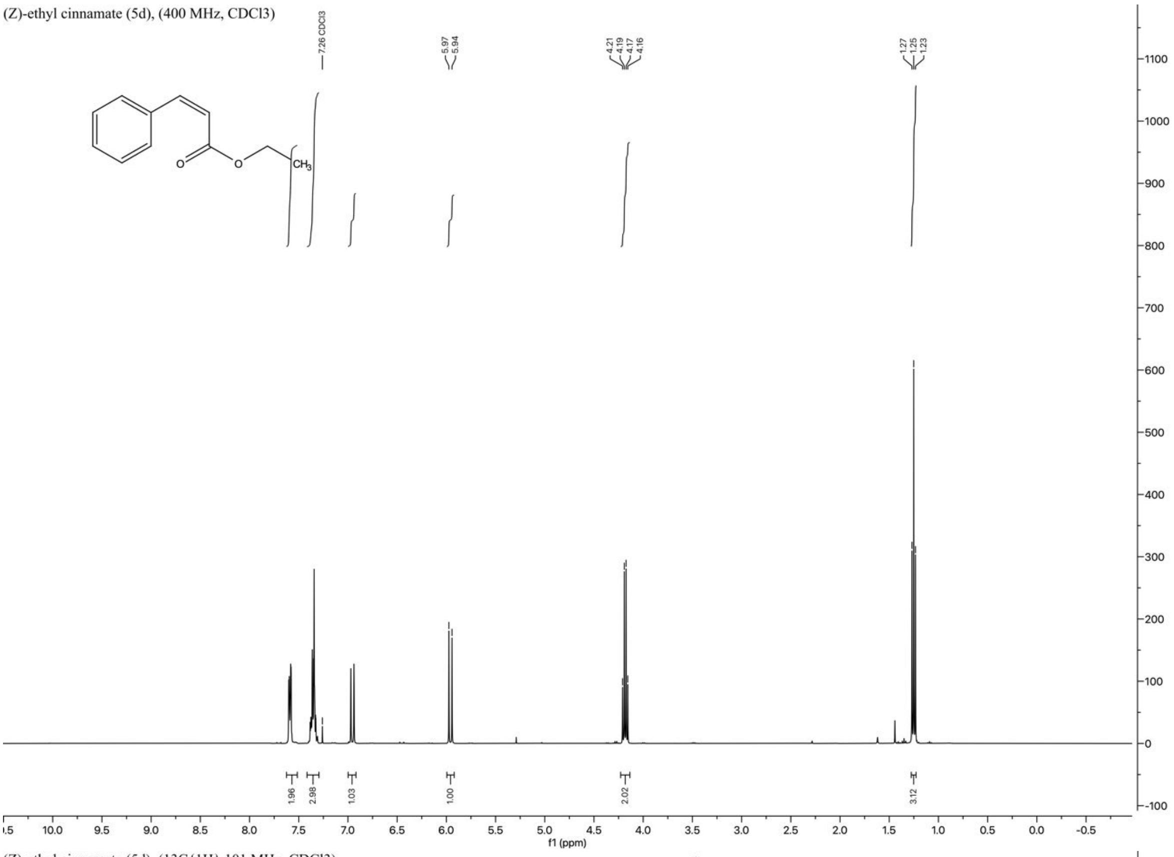

(Z)-ethyl cinnamate (5d), (13C{1H} 101 MHz, CDCl3)

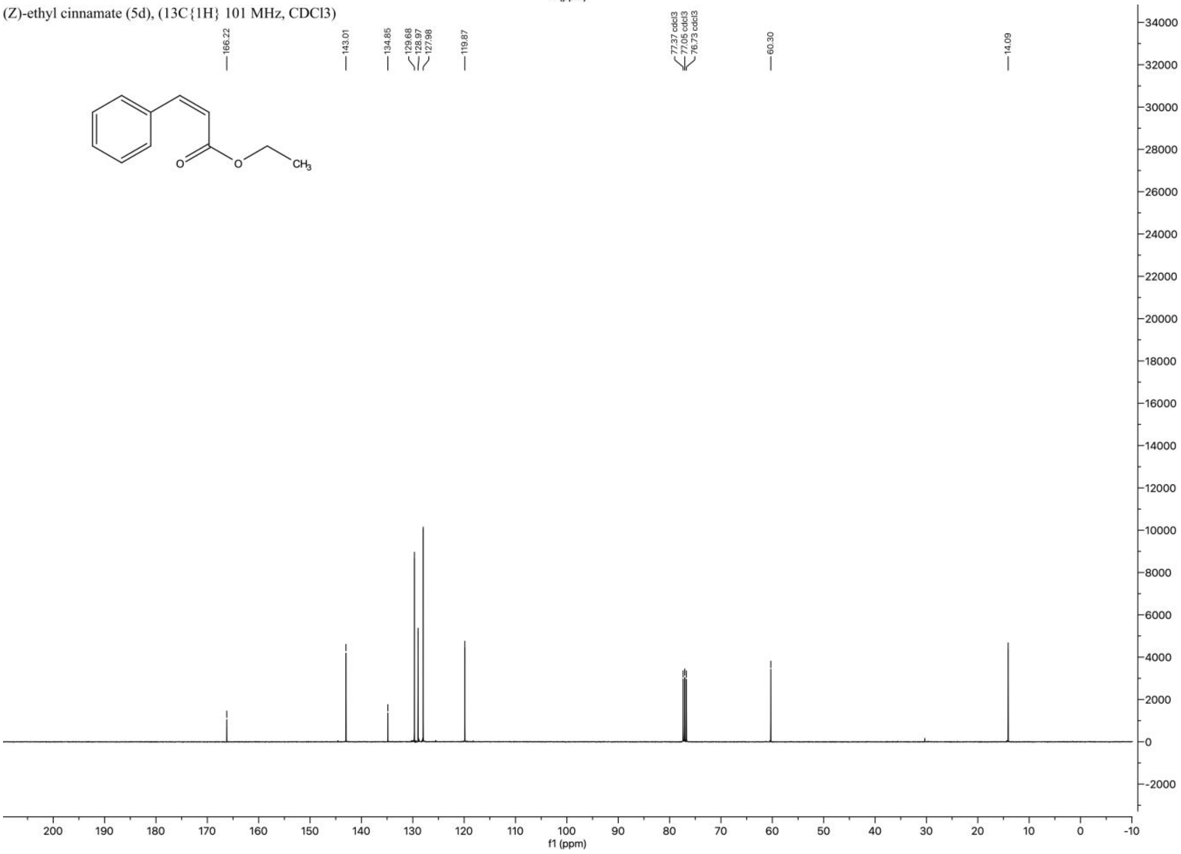

(Z)-cinnamaldehyde (5e), (400 MHz, CDCl3)

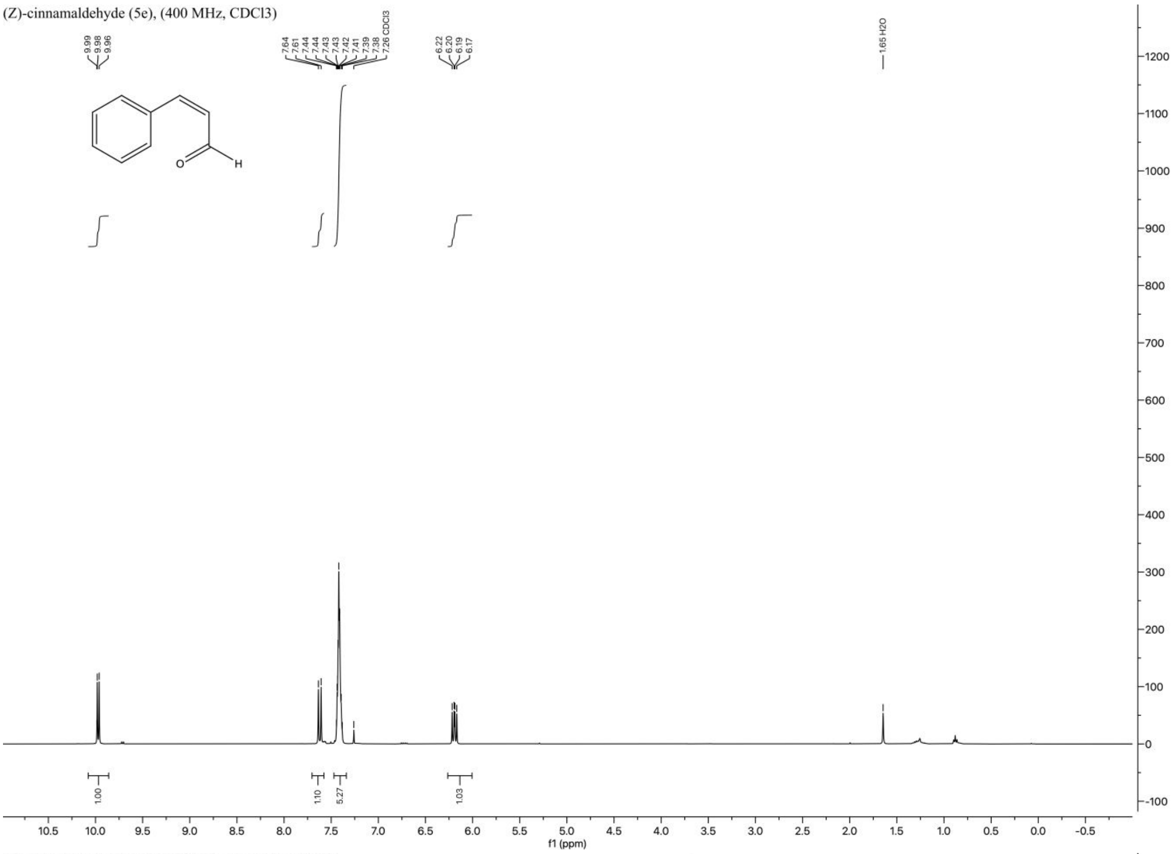

(Z)-cinnamaldehyde (5e), (13C{1H} 101 MHz, CDCl3)

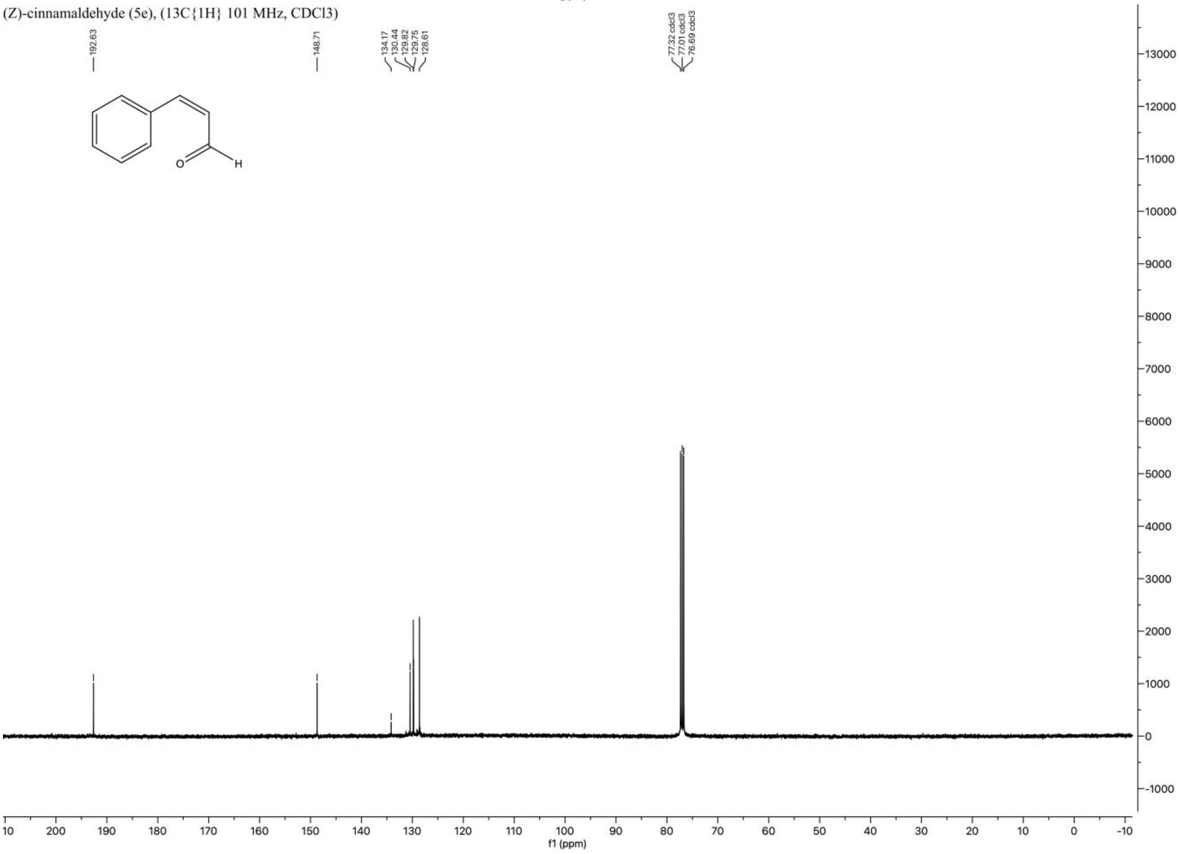

2H-chromen-2-one (5f), (400 MHz, CDCl3)

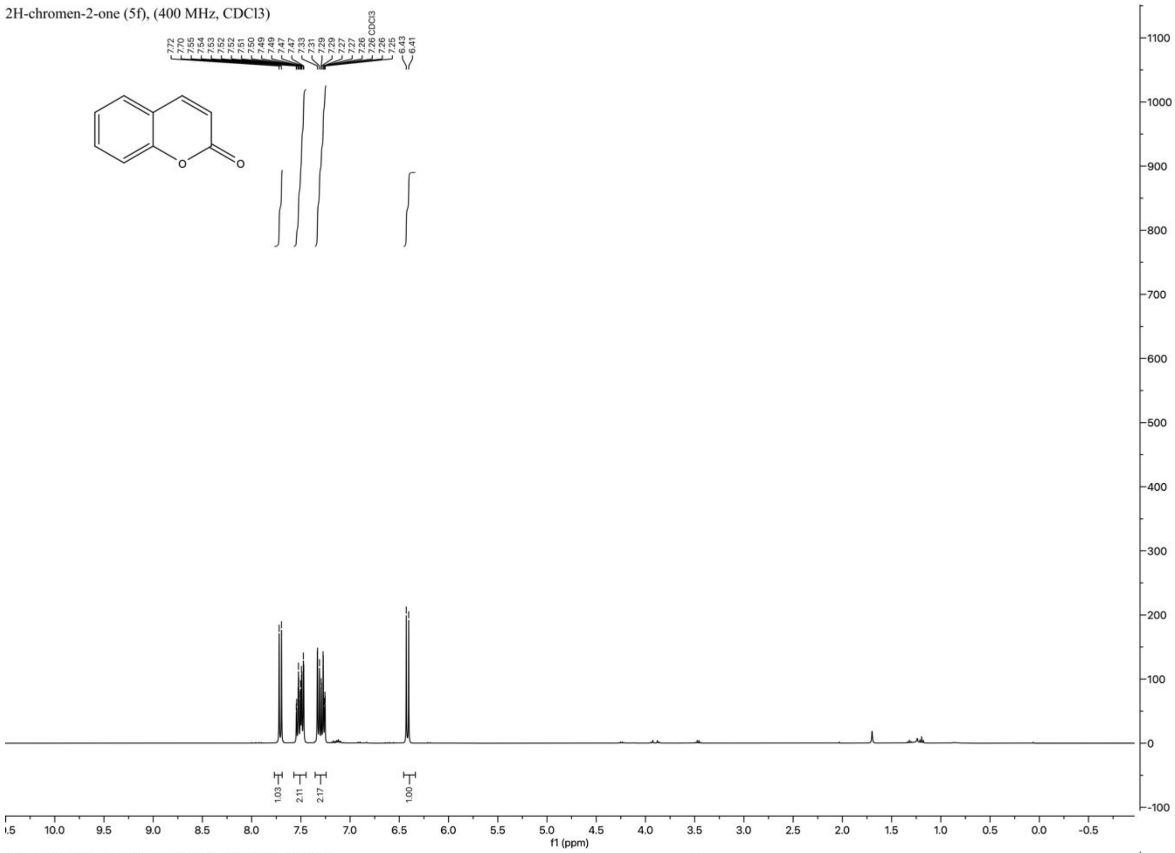

2H-chromen-2-one (5f), (13C{1H} 101 MHz, CDCl3)

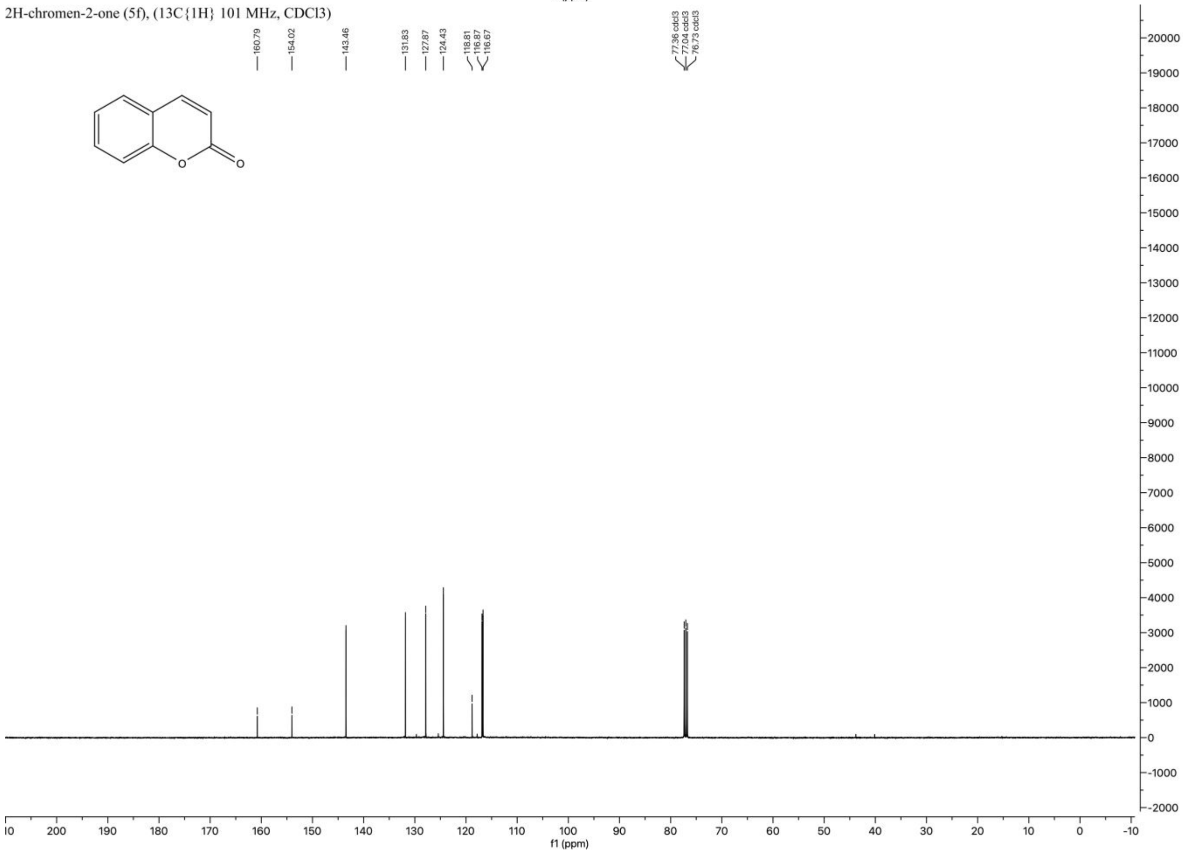

Supplement: Supplementary file 1 — gg2c00024_si_001.pdf [file gg2c00024_si_001.pdf]
